# Randomized phase 2 trial of pevonedistat plus azacitidine versus azacitidine for higher-risk MDS/CMML or low-blast AML

**DOI:** 10.1038/s41375-021-01125-4

**Published:** 2021-01-22

**Authors:** Mikkael A. Sekeres, Justin Watts, Atanas Radinoff, Montserrat Arnan Sangerman, Marco Cerrano, Patricia Font Lopez, Joshua F. Zeidner, Maria Diez Campelo, Carlos Graux, Jane Liesveld, Dominik Selleslag, Nikolay Tzvetkov, Robert J. Fram, Dan Zhao, Jill Bell, Sharon Friedlander, Douglas V. Faller, Lionel Adès

**Affiliations:** 1 Sylvester Comprehensive Cancer Center, University of Miami, Miami, FL USA; 2 University Hospital Sveti Ivan Rislki, Sofia, Bulgaria; 3 Institut Català d’Oncologia-Institut d’Investigació Biomèdica de Bellvitge (IDIBELL), Hospitalet, Barcelona, Spain; 4 Department of Molecular Biotechnology and Health Sciences, Division of Hematology, University of Turin, Turin, Italy; 5 Hospital General Universitario Gregorio Marañón, Instituto de Investigación Sanitaria Gregorio Marañón (IiSGM), Madrid, Spain; 6 University of North Carolina, Lineberger Comprehensive Cancer Center, Chapel Hill, NC USA; 7 University Hospital of Salamanca, IBSAL Institute for Biomedical Research of Salamanca, Salamanca, Spain; 8 Department of Hematology, Université Catholique de Louvain, CHU UCL Namur (Godinne site), Yvoir, Belgium; 9 The James P Wilmot Cancer Institute, University of Rochester, Rochester, NY USA; 10 AZ Sint Jan Brugge-Oostende, Brugge, Belgium; 11 MHAT Dr. Georgi Stranski, Clinic of Haematology, Pleven, Bulgaria; 12 Millennium Pharmaceuticals, Inc. a wholly owned subsidiary of Takeda Pharmaceutical Company Limited, Cambridge, MA USA; 13 AP-HP, Hôpital Saint Louis, Paris, France; 14 University of Paris, and INSERM U944, Paris, France

**Keywords:** Acute myeloid leukaemia, Randomized controlled trials, Myelodysplastic syndrome

## To the Editor

There is a critical unmet need for novel treatments for higher-risk myelodysplastic syndromes (MDS), higher-risk chronic myelomonocytic leukemia (CMML), and low-blast (LB) acute myeloid leukemia (AML). For patients ineligible for stem cell transplant (SCT), standard therapy with hypomethylating agents, such as azacitidine and decitabine, is not curative, with most patients relapsing within 2 years [1–3].

Pevonedistat is the first small-molecule inhibitor of the neural precursor cell expressed, developmentally downregulated 8 (NEDD8)-activating enzyme (NAE); NAE facilitates conjugation of the small ubiquitin-like protein, NEDD8, which activates cullin-RING E3 ubiquitin ligases (CRLs) [4–6]. Inhibition of NAE by pevonedistat prevents degradation of CRL substrates integral to tumor cell growth, proliferation, and survival, thereby leading to cancer cell death [4–6]. Pevonedistat + azacitidine demonstrated preclinical synergistic antitumor activity in AML xenografts and was well tolerated in patients with untreated AML, with promising clinical activity [7]. Based on these results, this phase 2, multicenter, global, randomized, controlled, open-label trial (NCT02610777) compared pevonedistat + azacitidine versus single-agent azacitidine in patients with higher-risk MDS/CMML and LB-AML who had not previously received a hypomethylating agent.

The study enrolled adults with morphologically confirmed higher-risk MDS, non-proliferative CMML, or LB-AML (20–30% myeloblasts in bone marrow); these patients were eligible for enrollment because the diseases are part of the higher-risk MDS spectrum, and were included in the pivotal randomized study that demonstrated significant improvement in overall survival (OS) with azacitidine versus conventional care regimens [3, 8, 9]. Patients with MDS/CMML were required to have very-high, high, or intermediate risk according to the revised international prognostic scoring system (IPSS-R); patients with intermediate-risk IPSS-R (>3–4.5 points) had ≥5% bone marrow myeloblasts (see Supplementary Appendix for detailed eligibility criteria).

Patients were randomized 1:1 to receive either pevonedistat 20 mg/m^2^ (intravenous) on days 1/3/5, plus azacitidine 75 mg/m^2^ (intravenous or subcutaneous) on days 1–5/8/9, or azacitidine alone on the same schedule, in 28-day cycles, and stratified into four categories: LB-AML, and MDS/CMML with IPSS-R risk of very-high/high/intermediate. Treatment continued until unacceptable toxicity, relapse, transformation to AML (defined according to World Health Organization classification as >20% blasts in blood or marrow and 50% increase in blast count from baseline [8]), progressive disease (PD), or the initiation of subsequent anticancer therapy or hematopoietic SCT. Patients with PD could continue treatment if deriving clinical benefit if their disease had not transformed to AML.

The study was initially powered for a primary endpoint of event-free survival (EFS; defined as the time from randomization to death or transformation to AML in higher-risk MDS/CMML, or death in LB-AML). In consultation with regulatory agencies following completion of enrollment, the primary endpoint was changed to OS, with EFS as a secondary endpoint. Other secondary and exploratory endpoints are listed in the Supplementary Appendix. Response assessment was based on modified international working group (IWG) criteria for MDS for patients with higher-risk MDS/CMML [10] and revised recommendations of the IWG for AML for patients with LB-AML [11]. Disease assessments were based on local bone marrow aspirate blast counts and transfusions, and central laboratory data. Toxicity was evaluated according to National Cancer Institute Common Terminology Criteria for Adverse Events, version 4.03. Further details of assessments and statistical analysis are provided in the Supplementary Appendix.

Overall, 120 patients from 45 sites in 12 countries were enrolled (pevonedistat + azacitidine: 58 patients; azacitidine: 62 patients) (Supplementary Fig. 1). Baseline demographics and disease characteristics were generally well-balanced between arms (Supplementary Table 1).

**Fig. 1:**
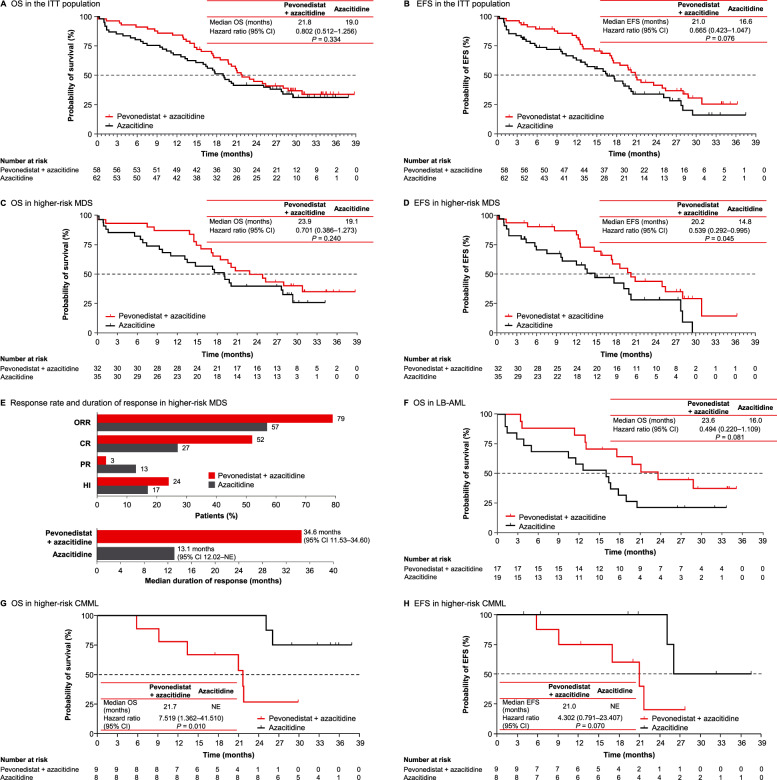
Overall survival, event-free survival, response rates, and response duration for study population and disease subgroups. **a** OS in the ITT population. **b** EFS in the ITT population. **c** OS in higher-risk MDS. **d** EFS in higher-risk MDS. **e** Response rate and duration of response in higher-risk MDS (**f**) OS in LB-AML. **g** OS in higher-risk CMML; **h** EFS in higher-risk CMML. CI confidence interval, CR complete remission, EFS event-free survival, HI hematologic improvement, ITT intent-to-treat, LB-AML low-blast AML, MDS myelodysplastic syndromes, NE not evaluable, ORR objective response rate, OS overall survival, PR partial response.

At data cutoff for the final analysis of this randomized proof-of-concept study, median follow-up was 21.4 and 19.0 months in the pevonedistat + azacitidine and azacitidine arms, respectively. In the intent-to-treat (ITT) population, pevonedistat + azacitidine demonstrated clinically meaningful increases in OS (median 21.8 months versus 19.0 months; *P* = 0.334; Fig. 1a), and EFS (median 21.0 versus 16.6 months; *P* = 0.076; Fig. 1b) compared with azacitidine alone. Among 108 response-evaluable patients, overall response rate (ORR; defined as complete remission [CR] + partial remission [PR] + hematologic improvement [HI] in higher-risk MDS/CMML, and CR + CR with incomplete blood count recovery [CRi] + PR in LB-AML) with pevonedistat + azacitidine versus azacitidine was 70.9% versus 60.4%, and the median duration of response was 20.6 months versus 13.1 months (Supplementary Table 2).

Improved efficacy outcomes were particularly pronounced in patients with higher-risk MDS. Median OS with pevonedistat + azacitidine versus azacitidine was 23.9 versus 19.1 months (Fig. 1c), and pevonedistat + azacitidine led to longer EFS compared with azacitidine (median 20.2 versus 14.8 months; HR: 0.539; *P* = 0.045; Fig. 1d). Patients with higher-risk MDS were more likely to achieve a response with pevonedistat + azacitidine versus azacitidine (ORR 79.3% versus 56.7%); the CR rate was nearly doubled (51.7% versus 26.7%) and duration of response was also improved (median 34.6 versus 13.1 months) (Fig. 1e).

Data on EFS and OS in prespecified subgroups, time to treatment failure (TTF), transformation to AML, transfusion independence, and subsequent SCT are available in Supplementary Appendix/Supplementary Figs. 2–6. In patients with higher-risk MDS, TTF was longer (median 19.7 versus 13.6 months; HR: 0.521; *P* = 0.025) and the rate of transfusion independence in patients with higher-risk MDS who were transfusion-dependent at baseline was higher with pevonedistat + azacitidine versus azacitidine alone (69.2% versus 47.4%; *P* = 0.228).

In LB-AML, median OS (equivalent to EFS) trended longer with pevonedistat + azacitidine versus azacitidine (23.6 versus 16.0 months, *P* = 0.081; Fig. 1f) although there was no ORR increase (52.9% versus 60.0%; CR/CRi 41.2% versus 60.0%). However, the LB-AML population was small (*n* = 32), and differences in baseline rate of AML with myelodysplasia-related changes (71% versus 42% with pevonedistat + azacitidine versus azacitidine) and differing proportions of patients with adverse risk according to European LeukemiaNet 2017 guidelines (59% versus 26%) may have affected response rates.

In higher-risk CMML, median OS was 21.7 months versus not evaluable (NE) (Fig. 1g), and median EFS was 21.0 months versus NE (Fig. 1h) with pevonedistat + azacitidine versus azacitidine, respectively. ORR was 77.8% with pevonedistat + azacitidine versus 75.0% with azacitidine (Supplementary Table 2). Although there was no observed benefit, the small number of patients with higher-risk CMML (17 total) precludes meaningful conclusions.

At data cutoff, patients in the pevonedistat + azacitidine arm had received a median of 13.0 cycles (range: 1–37) of pevonedistat and 13.0 cycles (range: 1–39) of azacitidine; patients in the azacitidine arm received a median of 8.5 cycles (range: 1–41) of azacitidine. The higher number of treatment cycles with pevonedistat + azacitidine compared with azacitidine alone was consistent with the observed longer duration of response in the combination arm. To determine if the slightly higher number of patients with very-high-risk (VHR)-MDS in the azacitidine arm (*n* = 16/35) versus the combination arm (*n* = 10/32) may have contributed to the potential benefit observed with pevonedistat + azacitidine, sensitivity analyzes for OS and EFS (statistical analysis details provided in the Supplementary Appendix) demonstrated that the treatment effect was maintained after stratification adjustment for IPSS-R risk category. Median pevonedistat dose intensity was 98.7%; median azacitidine dose intensity was similar between treatment arms (pevonedistat + azacitidine: 96.9%, azacitidine: 98.2%).

Overall, the safety profile of pevonedistat + azacitidine was comparable to that of azacitidine alone (Table 1). Grade ≥ 3 TEAEs were reported in 90% of patients with pevonedistat + azacitidine versus 87% with azacitidine. The most frequent grade ≥ 3 TEAEs were neutropenia (33% versus 27%), febrile neutropenia (26% versus 29%), anemia (19% versus 27%), and thrombocytopenia (19% versus 23%). The addition of pevonedistat to azacitidine did not result in additional myelosuppression, which is important for patients with disease- and age-related comorbidities and azacitidine dosing was not compromised. Consequently, patients could remain on treatment for longer with pevonedistat + azacitidine versus azacitidine alone. This contrasts with prior studies, in which the addition of a second agent to azacitidine led to increased toxicity, resulting in azacitidine dose reductions or shorter dosing schedules [12, 13]. On-study deaths occurred in 9% of pevonedistat + azacitidine-treated patients versus 16% with azacitidine. The 60-day mortality rate was 3.4% versus 12.9%; causes of death within 60 days included acute cardiac failure and multi-organ failure (both *n* = 1) with pevonedistat + azacitidine, and gastric necrosis, hypoxia, multiorgan failure, pneumonia, the progression of MDS, sepsis, subdural hematoma, and unknown factors (all *n* = 1) with azacitidine alone.

**Table 1:** Overall safety profile, and most common any-grade and grade ≥ 3 TEAEs occurring in ≥10% of patients.

	Pevonedistat + azacitidine *n* = 58	Azacitidine alone *n* = 62	Total *N* = 120
AEs, *n* (%)			
Any AE	57 (98)	62 (100)	119 (99)
Any drug-related AE	44 (76)	50 (81)	94 (78)
Any grade ≥ 3 AE	52 (90)	54 (87)	106 (88)
Any drug-related grade ≥3 AE	26 (45)	29 (47)	55 (46)
Any serious AE	40 (69)	39 (63)	79 (66)
Any drug-related serious AE	9 (16)	10 (16)	19 (16)
AE leading to discontinuation, *n* (%)	10 (17)	13 (21)	23 (19)
On-study deaths, *n* (%)	5 (9)	10 (16)	15 (13)
Most common any-grade AEs (≥10% of patients), *n* (%)			
Constipation	21 (36)	29 (47)	50 (42)
Nausea	20 (34)	28 (45)	48 (40)
Pyrexia	22 (38)	25 (40)	47 (39)
Anemia	18 (31)	28 (45)	46 (38)
Cough	22 (38)	21 (34)	43 (36)
Neutropenia	20 (34)	18 (29)	38 (32)
Fatigue	12 (21)	25 (40)	37 (31)
Diarrhea	19 (33)	17 (27)	36 (30)
Febrile neutropenia	15 (26)	18 (29)	33 (28)
Asthenia	17 (29)	12 (19)	29 (24)
Dyspnea	13 (22)	15 (24)	28 (23)
Thrombocytopenia	14 (24)	14 (23)	28 (23)
Vomiting	14 (24)	13 (21)	27 (23)
Decreased appetite	11 (19)	12 (19)	23 (19)
Edema peripheral	12 (21)	8 (13)	20 (17)
Epistaxis	13 (22)	6 (10)	19 (16)
Pneumonia	9 (16)	10 (16)	19 (16)
Back pain	10 (17)	8 (13)	18 (15)
Neutrophil count decreased	12 (21)	6 (10)	18 (15)
Arthralgia	5 (9)	12 (19)	17 (14)
Dizziness	8 (14)	8 (13)	16 (13)
Hypokalemia	4 (7)	11 (18)	15 (13)
Abdominal pain	4 (7)	10 (16)	14 (12)
Fall	7 (12)	7 (11)	14 (12)
Pain in extremity	10 (17)	4 (6)	14 (12)
Platelet count decreased	7 (12)	7 (11)	14 (12)
Insomnia	6 (10)	7 (11)	13 (11)
Headache	3 (5)	9 (15)	12 (10)
Most common grade ≥ 3 AEs (≥10% of patients), *n* (%)			
Neutropenia	19 (33)	17 (27)	36 (30)
Febrile neutropenia	15 (26)	18 (29)	33 (28)
Anemia	11 (19)	17 (27)	28 (23)
Thrombocytopenia	11 (19)	14 (23)	25 (21)
Neutrophil count decreased	12 (21)	6 (10)	18 (15)
Pneumonia	7 (12)	6 (10)	13 (11)

*AE* adverse event, *TEAE* treatment-emergent adverse event.

Treatment with pevonedistat + azacitidine or azacitidine alone was associated with similar patient-reported symptoms, functioning, and health-related quality of life (HRQoL) (Supplementary Appendix/Supplementary Fig. 7). Baseline mutational profiling data suggest that the numerically higher ORR observed with pevonedistat + azacitidine occurred across prognostic subgroups, including in patients harboring poor prognostic mutations (Supplementary Appendix/Supplementary Figs. 8–10).

In summary, this randomized, proof-of-concept phase 2 study demonstrated clinical efficacy with pevonedistat + azacitidine in patients with higher-risk MDS and LB-AML. The OS, EFS, and ORR benefits were particularly promising among patients with higher-risk MDS, as was the OS benefit in LB-AML. The addition of pevonedistat to azacitidine resulted in a comparable safety profile to azacitidine alone, no increased myelosuppression, and azacitidine dose intensity was maintained. The combination of azacitidine and pevonedistat appears less myelosuppressive than azacitidine and venetoclax and more applicable to outpatient treatment [14]. Given the encouraging clinical activity in combination with azacitidine, its novel mechanism of action, and its nonmyelosuppressive safety profile, pevonedistat may be an ideal combination partner with other agents, such as venetoclax, as the treatment landscape evolves.

## Supplementary information

Supplementary Material

Supplementary Figure 1

Supplementary Figure 2

Supplementary Figure 3

Supplementary Figure 4

Supplementary Figure 5

Supplementary Figure 6

Supplementary Figure 7

Supplementary Figure 8

Supplementary Figure 9

Supplementary Figure 10

Manuscript Video Summary

## Data Availability

The datasets, including the redacted study protocol, redacted statistical analysis plan, and individual participants data supporting the results reported in this article, will be made available within 3 months from initial request to researchers who provide a methodologically sound proposal. The data will be provided after its de-identification, in compliance with applicable privacy laws, data protection, and requirements for consent and anonymization.
