# Supplementary material for: Randomized phase 2 trial of pevonedistat plus azacitidine versus azacitidine for higher-risk MDS/CMML or low-blast AML
Source: Leukemia. 2021 Jan 22;35(7):2119–24. doi: 10.1038/s41375-021-01125-4 (PMC8257476; doi:10.1038/s41375-021-01125-4)
Supplement: Supplementary file 1 — Supplementary Material [file 41375_2021_1125_MOESM1_ESM.docx]

**Supplementary Appendix**

**Supplement to: Sekeres MA, et al. Randomized phase 2 trial of pevonedistat plus azacitidine versus azacitidine for higher-risk MDS/CMML or low-blast AML**

**Contents**

Additional Methodology page 2

Additional Results page 13

Supplemental Figures page 17

Supplemental Tables page 31

**Additional Methodology**

The trial complied with International Conference on Harmonization Good Clinical Practice guidelines and appropriate regulatory requirements. Independent ethics committees or institutional review boards approved the protocol. All patients provided written informed consent.

**Patient eligibility criteria**

**Inclusion criteria:**

Male or female patients 18 years or older

Morphologically confirmed diagnosis of myelodysplastic syndromes (MDS), nonproliferative chronic myelomonocytic leukemia (CMML; i.e., with white blood cell [WBC] <20 000/µl), or low-blast acute myeloid leukemia (LB-AML) based on one of the following:

French-American-British classifications:

Refractory anemia with excess blasts (defined as having 5% to 20% myeloblasts in the bone marrow)

CMML with 10% to 19% myeloblasts in the bone marrow and/or 5% to 19% blasts in the blood

OR

World Health Organization classifications:

Refractory anemia with excess blasts-1 (defined as having 5% to 9% myeloblasts in the bone marrow)

Refractory anemia with excess blasts-2 (defined as having 10% to 19% myeloblasts in the bone marrow and/or 5% to 19% blasts in the blood)

CMML-2 (defined as having 10% to 19% myeloblasts in the bone marrow and/or 5% to 19% blasts in the blood)

CMML-1 (although CMML-1 is defined as having <10% myeloblasts in the bone marrow and/or <5% blasts in the blood, these patients may enroll only if bone marrow blasts ≥5%)

World Health Organization-defined AML patients with 20% to 30% myeloblasts in the bone marrow (defined as “low-blast AML”) and ≤30% myeloblasts in peripheral blood who are considered by investigator to be appropriate for azacitidine-based therapy

For MDS and CMML patients, prognostic risk category, based on the Revised International Prognostic Scoring System of:

Very high (>6 points),

High (>4.5–6 points), or

Intermediate (>3–4.5 points): a patient determined to be in the Intermediate prognostic risk category was only allowable in the setting of ≥5% bone marrow myeloblasts

Eastern Cooperative Oncology Group performance status of 0 to 2

Clinical laboratory values within the following parameters:

Albumin >2.7 g/dl

Total bilirubin <upper limit of normal (ULN) except in patients with Gilbert’s syndrome. Patients with Gilbert’s syndrome could enroll if direct bilirubin ≤1.5 x ULN of the direct bilirubin

Alanine aminotransferase (ALT) and aspartate aminotransferase (AST) ≤2.5 × ULN

Creatinine clearance ≥50 ml/min

Hemoglobin >8 g/dl. Patients could be transfused to achieve this value. Elevated indirect bilirubin due to post-transfusion hemolysis was allowed

For CMML patients: WBC count <20 000/µl before administration of the first dose of study drug on cycle 1 cay 1; patients must have been off hydroxyurea for at least 1 week prior to WBC count assessment

Ability to undergo the study-required bone marrow sample collection procedures

Suitable venous access for the study-required blood sampling (i.e., including pharmacokinetics and biomarker sampling)

**Exclusion criteria:**

1. Previous treatment with decitabine or azacitidine or other hypomethylating agent
2. Acute promyelocytic leukemia as diagnosed by morphologic examination of bone marrow, by fluorescent in situ hybridization or cytogenetics of peripheral blood or bone marrow, or by other accepted analysis
3. Eligible for allogenic stem cell transplantation
4. Patients with MDS, CMML, or LB-AML whose only site of disease was extramedullary, e.g., the skin
5. Any serious medical or psychiatric illness that could, in the investigator’s opinion, potentially interfere with the completion of study procedures or could limit expected survival to less than 6 months
6. Treatment with any anti-leukemic/anti-MDS therapies (e.g., lenalidomide, cytarabine, anthracyclines, purine analogs) or with any investigational products within 14 days before the first dose of any study drug
7. Known hypersensitivity to mannitol
8. Active uncontrolled infection or severe infectious disease, such as severe pneumonia, meningitis, or septicemia
9. Major surgery within 14 days before first dose or a scheduled surgery during study period
10. Diagnosed or treated for another malignancy within 2 years before randomization or previously diagnosed with another malignancy and with any evidence of residual disease. Patients with nonmelanoma skin cancer or carcinoma in situ of any type were not excluded if they had undergone resection
11. Life-threatening illness unrelated to cancer
12. Prothrombin time or activated partial thromboplastin time, >1.5 ULN or active uncontrolled coagulopathy or bleeding disorder
13. Known human immunodeficiency virus seropositive
14. Known hepatitis B surface antigen seropositive, or known or suspected active hepatitis C infection
15. Known hepatic cirrhosis or severe pre-existing hepatic impairment
16. Known cardiopulmonary disease defined as unstable angina, clinically significant arrhythmia, congestive heart failure (New York Heart Association Class III or IV), and/or myocardial infarction within 6 months prior to first dose, or severe pulmonary hypertension
17. Treatment with strong CYP3A inhibitors or inducers within 14 days before the first dose of pevonedistat
18. Systemic antineoplastic therapy or radiotherapy for other malignant conditions within 12 months before the first dose of any study drug, except for hydroxyurea

**Pevonedistat administration**

Pevonedistat was administered via a 60-minute intravenous infusion on days 1, 3, and 5 of each 28-day cycle, after the subcutaneous or intravenous azacitidine dose. The infusion of pevonedistat began between 15 and 60 minutes after completion of administration of the subcutaneous azacitidine dose or between 30 and 60 minutes after completion of intravenous azacitidine, which may be inconvenient for some patients.

**Azacitidine dose modification guidelines**

**Hematologic toxicity**

- Dose reduction or delays of azacitidine for hematologic toxicities (including fever and neutropenia) during the first 6 cycles of therapy were strongly discouraged, as it may impact patient outcome
- For hematologic adverse events, the start of a new treatment cycle was to be delayed and/or dose modifications considered if:
  - Absolute neutrophil count (ANC) was <500/µl. For patients with disease-related neutropenia, physician discretion could be used to initiate therapy with ANC ≥50% of baseline (baseline from the start of the previous cycle). In general, the use of growth factors was restricted. However, to avoid dose delay, patients who experienced grade 4 neutropenia (ANC <500/µl) with or without fever could receive granulocyte colony-stimulating factor or granulocyte macrophage colony-stimulating factor between days 28 to 42 of azacitidine monotherapy or combination after discussion and agreement with the sponsor’s project clinician (or designee). Patients who received myeloid growth factors were not included in assessment of neutrophil response
  - Platelet count was <20 000/µl. For patients with disease-related thrombocytopenia, physician discretion could be used to initiate therapy with platelet count ≥50% of baseline (baseline from the start of the previous cycle). If the above criteria were not met, the start of the new cycle was delayed until the above criteria were met. If a low ANC or platelet count caused the delay of the start of the new cycle of more than 2 weeks, then the azacitidine dose was decreased to 50 mg/m^2^ when treatment was resumed. Treatment could be held up to 6 weeks (42 days) due to toxicity before the patient had to be removed from protocol therapy

**Non-hematologic toxicity**

- For renal toxicities, specifically elevated creatinine >grade 1, azacitidine was reduced in accordance with the prescribing information and/or institutional guidelines
- Similarly, if unexplained elevations in serum creatinine or blood urea nitrogen occurred, the next cycle was delayed until values returned to normal or baseline values, and the dose was reduced by 50% on the next treatment course. If unexplained reductions in serum bicarbonate levels to <20 mEq/l occurred, the azacitidine dose was reduced by 50% on the next course

**Pevonedistat dose modification guidelines**

**Hematologic toxicity**

- If clinically indicated in the opinion of the investigator, the pevonedistat dose could be reduced from 20 mg/m^2^ to 10 mg/m^2^. The pevonedistat dose could be re-escalated to 15 mg/m^2^ or 20 mg/m^2^ at the next cycle, if the toxicity had recovered to ≤grade 1 or the patient’s baseline
- Pevonedistat was to be held for symptoms of leukostasis until the leukostasis was treated per institutional guidelines. Pevonedistat could be restarted when WBC count was <50 000/µl

**Serum transaminases and total bilirubin**

- For elevated liver function tests of grade 2 or 3 that occurred on or after cycle 1 day 3, pevonedistat was held; once the elevated AST or ALT returned to ≤grade 1, and/or elevated bilirubin returned to ≤1.5 x ULN or the patient’s baseline level, pevonedistat dose could be resumed at 20 mg/m^2^. For pevonedistat, a minimum of 1 full calendar day between any two doses was be maintained, and a maximum of three doses of pevonedistat within the cycle was not exceeded
- For elevated liver function tests of grade 4 that occurred on or after cycle 1 day 3, the pevonedistat dose was be held for the remainder of the cycle; if the elevated AST or ALT returned to ≤grade 1, and/or elevated bilirubin returned to ≤1.5 x ULN or the patient’s baseline level, then pevonedistat could be restarted at the next cycle at a reduced dose of 10 mg/m^2^. If the toxicity returned to ≤grade 1 or the patient’s baseline status, pevonedistat could be re-escalated to 15 mg/m^2^ for the next cycle. After 1 cycle at 15 mg/m^2^, further re-escalation to 20 mg/m^2^ could occur only after the patient’s liver function test (AST, ALT, and bilirubin) were confirmed to be ≤grade 1, the same level as the patient’s baseline values

**Hypophosphatemia**

- If hypophosphatemia was ≥grade 3, study drug treatment was not resumed until the hypophosphatemia was ≤grade 2

**Other toxicities**

- For other ≥grade 2 nonhematologic toxicities potentially related to pevonedistat, the pevonedistat dose could be reduced from 20 mg/m^2^ to 10 mg/m^2^ at the discretion of the investigator as clinically indicated. If the toxicity returned to ≤grade 1 or the patient’s baseline status, pevonedistat could be re-escalated to 15 mg/m^2^ or 20 mg/m^2^ at the next cycle

**Randomization and stratification**

Randomization and stratification were conducted centrally using an interactive web response system, and patients were randomized sequentially at a center as they became eligible for randomization.

**Secondary endpoints**

- Time to AML transformation in higher-risk MDS/CMML
- Time to treatment failure (TTF; defined as time from randomization to PD, relapse, or death)
- Overall response rate (ORR; defined as complete remission [CR] + partial remission [PR] + hematologic improvement [HI] in higher-risk MDS/CMML, and CR + CR with incomplete blood count recovery [CRi] + PR in LB-AML);
- Treatment-emergent adverse events (TEAEs) and serious AEs
- Six-month and 1-year survival rates
- Time to first complete remission ([CR] for hazard ratio MDS/CMML and LB-AML; CR + CR with incomplete blood count recovery [composite CR] for LB-AML) or partial remission for hazard ratio MDS/CMML and LB-AML
- Time to subsequent therapy
- Red blood cell (RBC) and platelet-transfusion independence
- Abnormal clinical laboratory values, Eastern Cooperative Oncology Group performance status, electrocardiograms, and vital sign measurements

**Exploratory endpoints**

- Bone marrow blast reduction by cycle 2 and cycle 4
- Identification of cytogenetic abnormalities, somatic mutations, circulating proteins and metabolites, miRNA profiles at baseline, and gene expression and epigenetic changes between baseline and specified post-dose time point(s)
- Rate of cytogenetic CR among patients with CR or CR with incomplete blood count recovery
- Measurable residual disease status in patients who have reached CR at cycle 4 or cycle 7
- Association of polymorphic variants in rug metabolizing enzymes or transporters, as applicable, with pevonedistat exposure and/or safety
- Identification of efficacy or safety biomarkers by evaluating germline polymorphisms (such as in proteasome related genes)
- Identification of treatment-emergent resistance mechanisms, such as somatic mutations in NEDD8-activating enzyme subunits and key signaling pathways, or change in pathways activity, in tumors from patients who initially respond to therapy and then exhibit progressive disease
- Health-related quality of life assessed via: the European Organisation for the Research and Treatment of Cancer Core Quality of Life Questionnaire (EORTC-QLQ-C30), the Quality of Life in Hematological patients (myelodysplastic syndromes) questionnaire from the Associazione QOL-ONE (QOL-E; US patients only), and the EuroQoL 5 dimensions 5 levels (EQ-5D-5L) questionnaire.
- Correlation between event-free survival and overall survival endpoints in the intent-to-treat population and the disease subpopulations

**Assessments**

Bone marrow aspirates (BMAs) for disease assessments were performed on day 22 (+6 days) of cycles 2 and 4, and then as clinically indicated in patients who achieved a CR, and/or after completion of every third treatment cycle.

Among patients who were RBC and platelet transfusion-dependent at baseline, RBC and platelet transfusion-independence was defined as having no transfusion of RBCs or platelets for a period of ≥8 weeks after initiation of dosing with duration defined as the maximum time interval >8 weeks in which a patient had no RBC or platelet transfusions from start of treatment through 30 days after the last dose of study drug.

Assessment of patient-reported overall HRQoL and health status was conducted using the EORTC-QLQ-C30 and the EQ-5D-5L instruments, respectively. These assessments were completed by patients at screening, the start of every cycle, and the end of treatment (EOT), and then monthly during EFS or response follow-up until progression.

The EORTC QLQ-C30 assessed important HRQoL domains, including global health status/quality of life, functioning, and symptoms over the previous week, whereas the QOL-E, which was limited to patients in the United States, was used to assess the specific impact of MDS on HRQoL that is not measured in general non-MDS-specific instruments. Future attempts to assess patient-reported outcomes in this population could include the MDS-specific Quality of Life in Myelodysplasia Scale (QUALMS) instrument, which has now been validated and used in clinical trial settings.

Mutational analysis was conducted on BMA samples collected at screening. DNA sequencing was performed using a 573 TruSeq Hybrid Capture panel of frequently mutated genes in AML and MDS. Samples were sequenced with a mean target coverage of 9 500x. Single-nucleotide variants and insertions and deletions (indels) were identified with Mutect. A gene was designated “positive” for mutations if it had ≥1 nucleotide variant/s. FMS-like tyrosine kinase 3–internal tandem duplication mutations were further confirmed by polymerase chain reaction using the LeukoStrat® CDx test.

**Statistical analysis**

The study was initially powered based on randomization of 117 patients for a primary endpoint of EFS. In consultation with regulatory agencies following completion of study enrollment, the primary endpoint was changed to OS and was to be analyzed after 60% of OS events had occurred in the higher-risk MDS/CMML population; the study was therefore not powered for OS in the intent-to-treat (ITT) population with the initially planned number of patients. From then until the final analysis, the sponsor remained blinded to efficacy results.

OS was calculated from date of randomization to death due to any cause. EFS was calculated from the date of randomization to occurrence of an event. Allotransplanted patients were not censored at time of transplant for OS and EFS. Unstratified log-rank tests were used to compare OS and EFS between treatment arms. HRs and two-sided 95% CIs were estimated using unadjusted unstratified Cox models. Kaplan-Meier methodology was used to estimate time-to-event distributions. Sensitivity analyses for OS and EFS used stratified Cox regression model with treatment as a factor to estimate HRs plus 95% CIs and stratified log-rank test to calculate p-values.

The analysis of the primary endpoint and all secondary efficacy endpoints that were not response-related was based on the intent-to-treat population, defined as all randomized patients. The response-evaluable population was defined as patients who received at least 1 dose of study drug, had a disease assessment at baseline, and at least 1 post-baseline disease assessment. The safety population was defined as all patients who received at least 1 dose of study drug.

Two interim analyses were planned for this study. The first interim analysis was planned for safety, based on 60-day mortality. When 60 patients had been on study for 60 days, safety and efficacy data were reviewed by the independent data monitoring committee, who recommended continuation of the study. The second interim analysis was to evaluate both efficacy and safety data when approximately 23 event-free survival events had occurred. At a median follow up of 8.0 months for pevonedistat+azacitidine and 6.8 months for azacitidine, 23 event-free survival events had occurred, and the independent data monitoring committee recommended that the study continue as planned. The final analysis was performed when 60% of patients with higher-risk MDS/CMML had an overall survival event.

**Additional Results**

**Efficacy**

**OS and EFS in prespecified subgroups**

Analysis of OS in the ITT and higher-risk MDS populations by prespecified subgroups demonstrated a nonsignificant benefit with pevonedistat+azacitidine versus azacitidine alone in patients with IPSS-R very-high-risk MDS/CMML and higher risk MDS (Supplementary Fig. 2 and 3, respectively).

Analysis of EFS in the ITT and higher-risk MDS populations in prespecified subgroups favored pevonedistat+azacitidine versus azacitidine alone in patients with IPSS-R very-high-risk MDS/CMML and very-high-risk and high-risk MDS (Supplementary Fig. 4 and 5, respectively).

Kaplan-Meier curves for OS and EFS in higher-risk MDS patient subgroups with IPSS-R very-high-, high- and intermediate-risk are shown in Supplementary Fig. 6. Given the small numbers of patients in the IPSS-R subpopulations, statistical significance cannot be adequately assessed.

**Time to treatment failure**

In the ITT population, median TTF was 15.2 months with pevonedistat+azacitidine versus 13.6 months with azacitidine alone (HR: 0.790, 95% CI: 0.521–1.198; P = 0.266). TTF was longer with pevonedistat+azacitidine versus azacitidine alone among patients with higher-risk MDS (median 19.7 vs. 13.6 months; HR: 0.521, 95% CI: 0.291–0.931; P = 0.025) and trended longer in patients with LB-AML (median, 12.8 vs. 9.1 months; HR: 0.490, 95% CI: 0.225–1.067; P = 0.067). In patients with higher-risk CMML, median TTF was 13.2 months versus NE (HR: 6.680, 95% CI: 1.364–32.706; P = 0.008).

**Transformation to AML**

For patients with higher-risk MDS/CMML, median time to transformation to AML was NE with both pevonedistat+azacitidine and azacitidine alone (six vs. nine events; HR: 0.562, 95% CI: 0.200–1.579; P = 0.267).

**Transfusion independence**

In the ITT population, of the 26 patients receiving pevonedistat+azacitidine and 30 patients receiving azacitidine alone who were platelet or RBC transfusion-dependent at baseline, 69.2% and 50.0% became transfusion-independent, respectively (relative risk [RR]: 1.385, 95% CI: 0.892–2.150; P = 0.148). Median transfusion rate (number of RBC or platelet transfusions per month) in the ITT population was 1.6 and 1.9 in the pevonedistat+azacitidine and azacitidine alone arms, respectively. Among patients with higher-risk MDS who were RBC transfusion-dependent at baseline (pevonedistat+azacitidine: n = 13; azacitidine: n = 18), 69.2% versus 50.0% became transfusion-independent, and the median transfusion rate was 0.7 with pevonedistat+azacitidine versus 1.3 with azacitidine alone. Median duration of RBC transfusion-independence among patients with higher-risk MDS was 22.5 versus 11.6 months with pevonedistat+azacitidine versus azacitidine alone (HR: 0.225; P = 0.052).

**Subsequent allogeneic stem cell transplant**

After dosing with study drug, 16 patients subsequently received an allogeneic stem cell transplant (pevonedistat+azacitidine: n = 6; azacitidine alone: n = 10). The 16 patients who were transplanted were not initially eligible for transplantation; these patients improved clinically to the extent that their physicians felt they could benefit from transplantation (10 of 16 patients had CRs prior to transplantation).

**Treatment discontinuation**

Treatment was discontinued in 88% versus 90% of patients in the pevonedistat+azacitidine versus azacitidine arms; the primary reasons for discontinuation were adverse events (AEs; 17% versus 23%), and PD (38% versus 27%).

**Patient-reported outcomes**

No difference was observed in patient-reported HRQoL with pevonedistat+azacitidine versus azacitidine alone according to EORTC-QLQ-C30, with similar mean Global Health Status scores maintained from study entry to EOT (Supplementary Fig. 7). Analyses of other functioning and symptom subscales also showed similar scores between arms and preservation of HRQoL from baseline (data not shown). The mean EQ-5D-5L index and dimensions visual analogue scale scores between arms did not differ during the treatment period (data not shown).

Improvement in HRQoL has been notoriously difficult to demonstrate in MDS, and particularly in higher-risk MDS, as HRQoL in MDS is driven largely by symptoms of anemia. This was mitigated in both treatment arms, either by an HI response, or by blood transfusions.

**Molecular analysis**

Next generation sequencing analyses were conducted on 96 screening BMA samples of which 88 were from response-evaluable patients. Mutation frequencies in this study – compared with ranges of published frequencies of mutations in AML, MDS, and CMML – are shown in Supplementary Fig. 8. The observed mutation frequencies were consistent with published data for MDS, CMML, and AML, and the representation of the observed mutations was mostly balanced between arms.

Responses in higher-risk MDS and LB-AML with pevonedistat+azacitidine were observed in patients harboring poor prognostic mutations, such as mutations in TP53, DNMT3A, ASXL1, and RUNX1 (Supplementary Fig. 9). ORR was further analyzed by study arm in higher-risk MDS patients harboring poor prognostic mutations (Supplementary Fig. 10). While the study was not powered for comparison of ORR according to mutational status, results reflected findings in the response-evaluable higher-risk MDS population, with numerically higher ORR in the pevonedistat+azacitidine arm. There were insufficient patients in the LB-AML and CMML cohorts to compare ORR in patients harboring poor prognostic mutations between study arms.

**Supplementary figures**

**Supplementary Fig. 1 CONSORT diagram.**


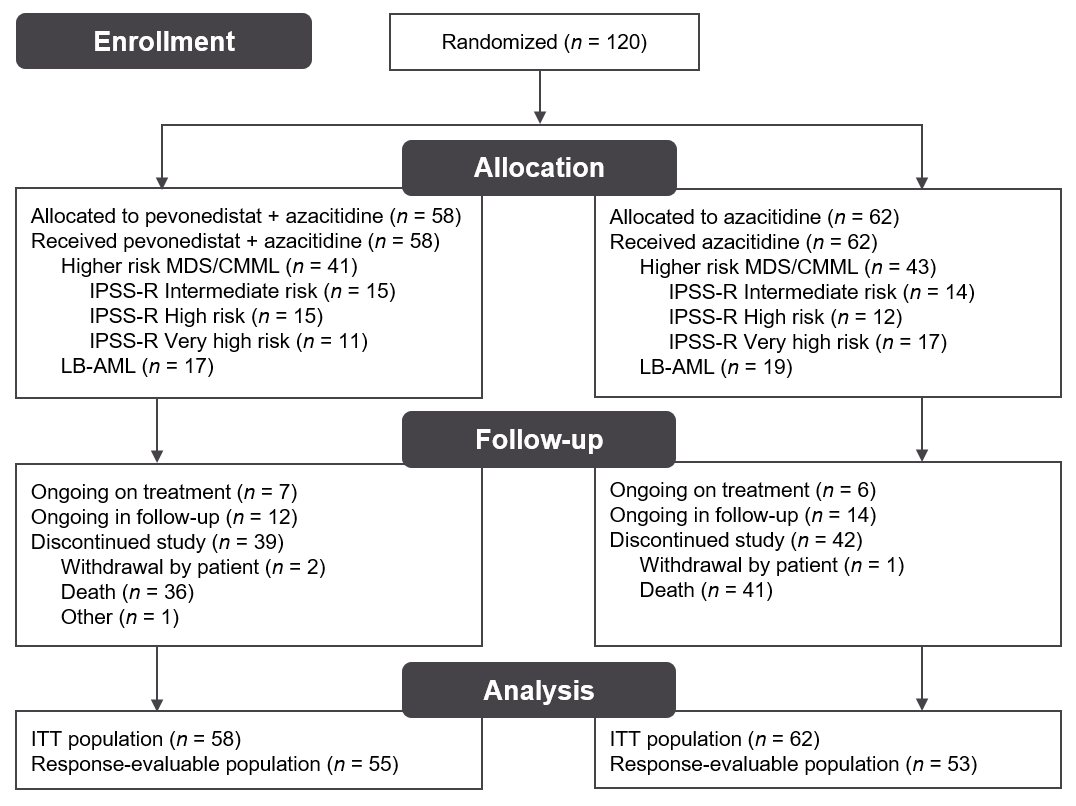


*CMML* chronic myelomonocytic leukemia, *IPSS-R* Revised International Prognostic Scoring System, *ITT* intent-to-treat, *LB-AML* low-blast acute myeloid leukemia, *MDS* myelodysplastic syndromes.

**Supplementary Fig. 2 Overall survival in prespecified patient subgroups in the intent-to-treat population.**


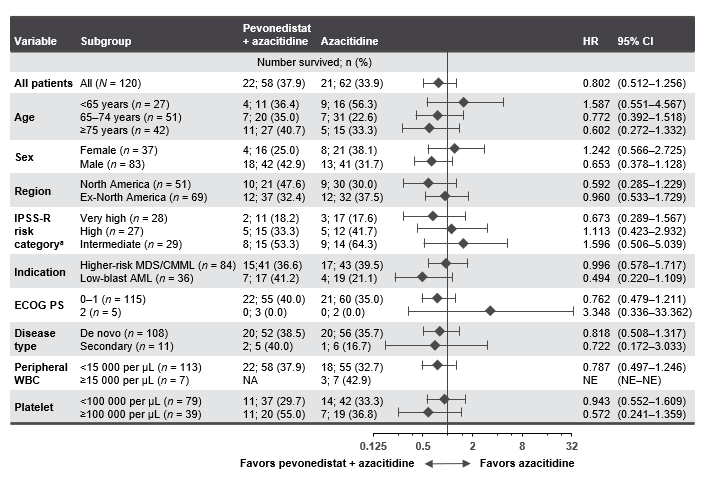


*AML* acute myeloid leukemia, *CI* confidence interval, *CMML* chronic myelomonocytic leukemia, *ECOG PS* Eastern Cooperate Oncology Group performance status, *HR* hazard ratio, *IPSS-R* Revised International Prognostic Scoring System, *MDS*, myelodysplastic syndromes, *NA* not applicable, *NE* not evaluable, WBC white blood cell. ^a^IPSS-R risk category pertains to patients with higher-risk MDS/CMML.

**Supplementary Fig. 3 Overall survival in prespecified patient subgroups in patients with higher-risk myelodysplastic syndromes.**


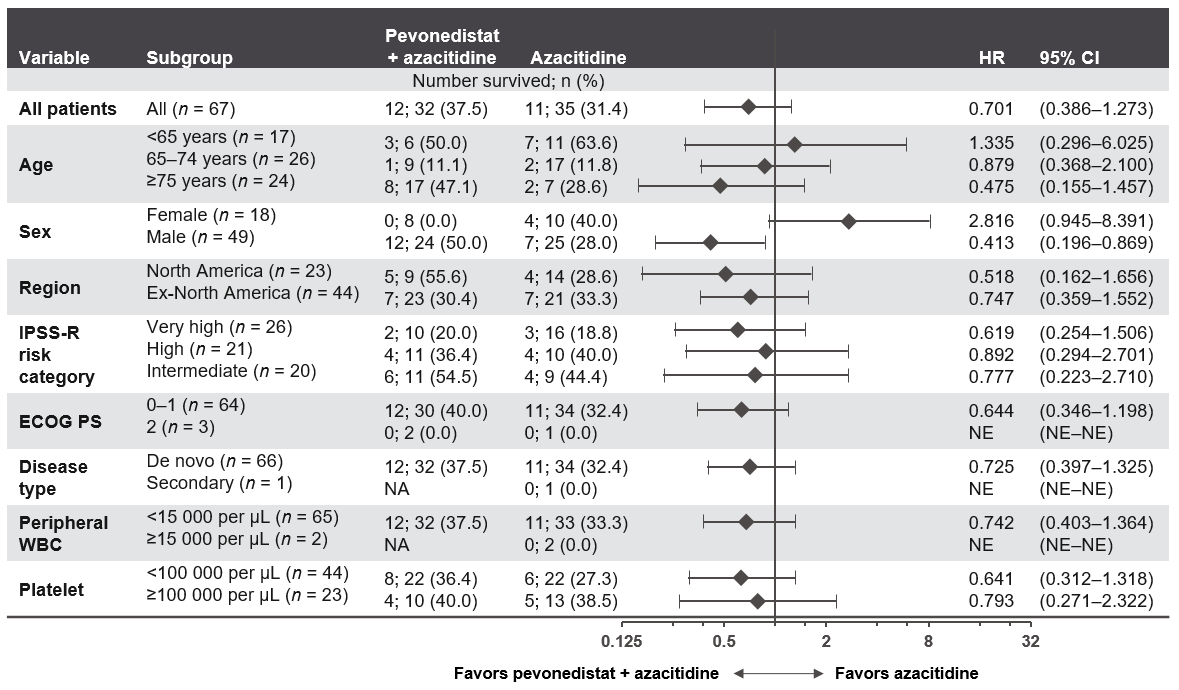


*CI* confidence interval, *ECOG PS* Eastern Cooperate Oncology Group performance status, *HR* hazard ratio, *IPSS-R* Revised International Prognostic Scoring System, *NA* not applicable, *NE* not evaluable, *WBC* white blood cell.

**Supplementary Fig. 4 Event-free survival in prespecified patient subgroups in the intent-to-treat population.**


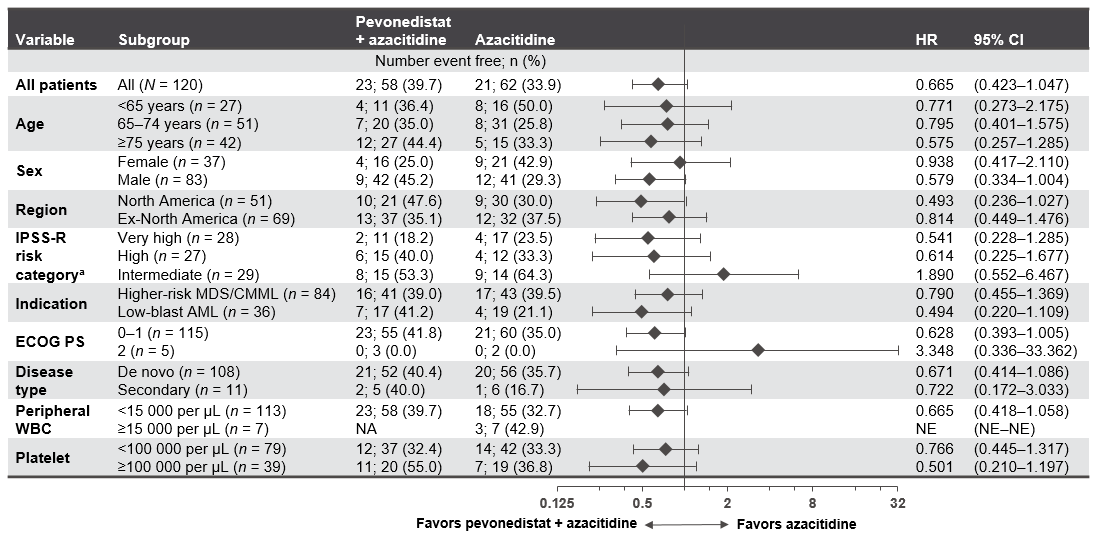


*AML* acute myeloid leukemia, *CI* confidence interval, *CMML* chronic myelomonocytic leukemia, *ECOG PS* Eastern Cooperate Oncology Group performance status, *HR* hazard ratio, *IPSS-R* Revised International Prognostic Scoring System, *MDS* myelodysplastic syndromes, *NA* not applicable, *NE* not evaluable, *WBC* white blood cell. ^a^IPSS-R risk category pertains to patients with HR MDS/CMML.

**Supplementary Fig. 5 Event-free survival in prespecified patient subgroups in patients with higher-risk myelodysplastic syndromes.**


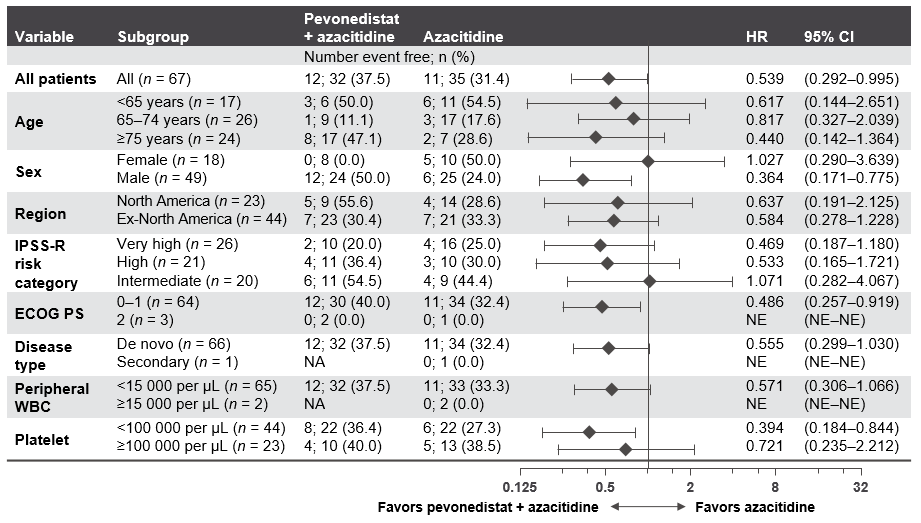


*CI* confidence interval, *ECOG PS* Eastern Cooperate Oncology Group performance status, *HR* hazard ratio, *IPSS-R* Revised International Prognostic Scoring System, *NA* not applicable, *NE* not evaluable, *WBC* white blood cell.

**Supplementary Fig. 6 OS and EFS in patients with higher-risk MDS with very high, high, or intermediate IPSS-R risk: (A) OS, very high IPSS-R risk (n=26); (B) EFS, very high IPSS-R risk (n=26); (C) OS, high IPSS-R risk (n=21); (D) EFS, high IPSS-R risk (n=21); (E) OS, intermediate IPSS-R risk (n=20) (F) EFS, intermediate IPSS-R risk (n=20)**


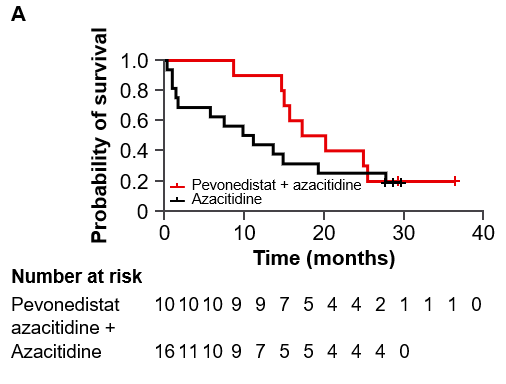


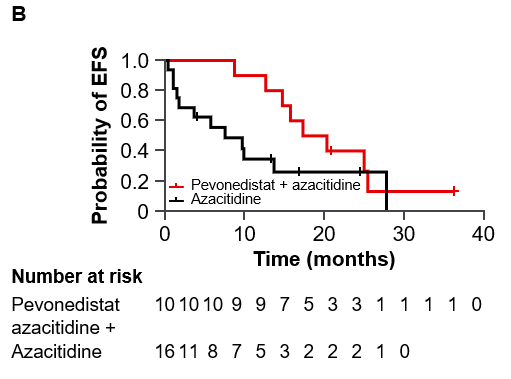


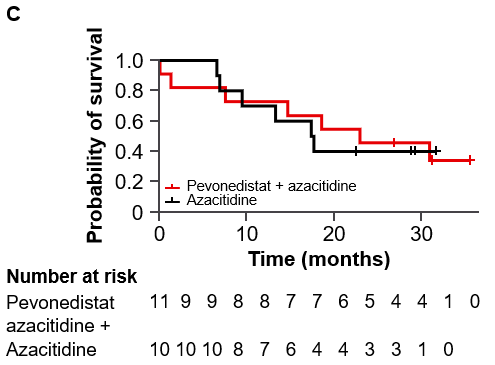


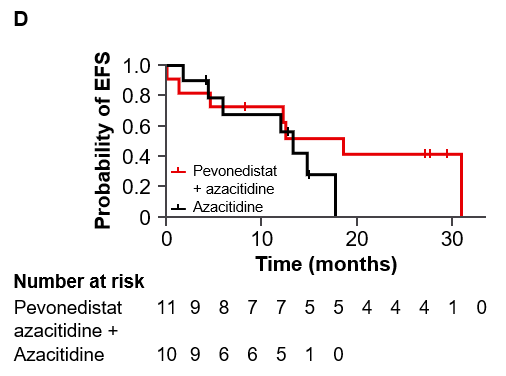


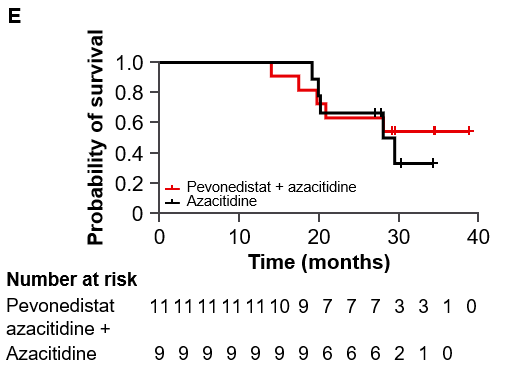


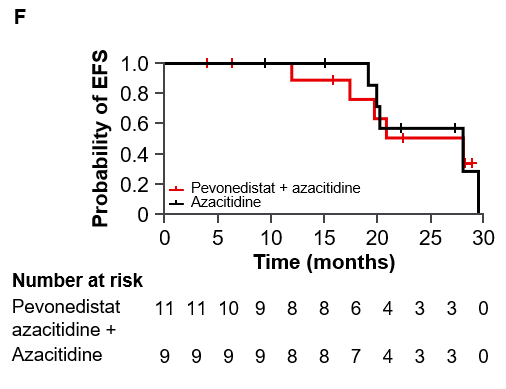


**Supplementary Fig. 7 Global health status (European Organization for Research and Treatment of Cancer Quality of Life Questionnaire-C30) mean score over time.**


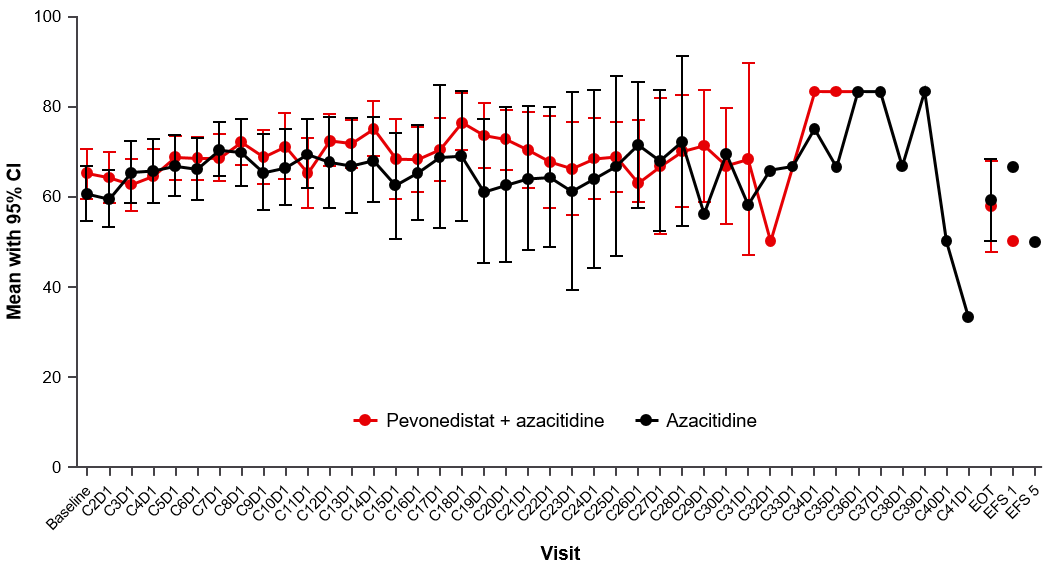


*C* cycle, *CI* confidence interval, *D* day, *EFS* event-free survival, *EOT* end of treatment.

**Supplementary Fig. 8 Mutation frequencies in study patients in comparison to published frequency ranges per indication.**

96 screening bone marrow aspirate samples were subjected to DNASeq (55x MDS; 15x CMML; 26x AML). FLT3-ITD mutations were further confirmed by polymerase chain reaction. Mutation frequencies were determined and compared to published frequency ranges of mutations,^a^ which generally have a frequency of at least 5% in MDS, CMML, and AML. Bars; range of mutation frequencies in MDS, CMML, and AML landscape, circles; mutation frequencies in the P-2001 study.


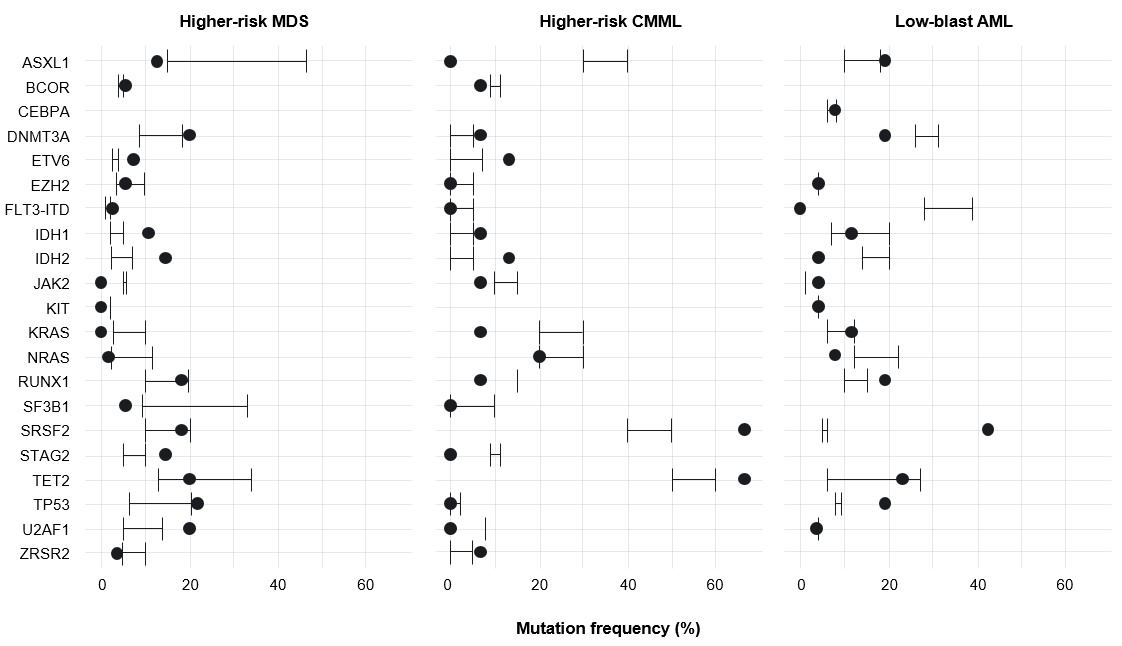


*AML* acute myeloid leukemia, *CMML* chronic myelomonocytic leukemia, *MDS* myelodysplastic syndromes.

^a^References for published mutation frequencies are as follows:

**MDS:**

1. Haferlach T, Nagata Y, Ogawa S *et al*. Landscape of genetic lesions in 944 patients with myelodysplastic syndromes. *Leukemia* 2014; **28**: 241–247.
2. Bejar R, Stevenson KE, Caughey B, Coleman Lindsley R, Mar BG, Stojanov P *et al*. Somatic mutations predict poor outcome in patients with myelodysplastic syndrome after hematopoietic stem-cell transplantation. *J Clin Oncol* 2014; **32**: 2691–2698.
3. Papaemmanuil E, Gerstung M, Malcovati L, Tauro S, Gundem G, Van Loo P *et al*. Clinical and biological implications of driver mutations in myelodysplastic syndromes. *Blood* 2013; **122**: 3616–3627.
4. Harada H, Harada Y. Recent advances in myelodysplastic syndromes: molecular pathogenesis and its implications for targeted therapies. *Cancer Sci* 2015; **106**: 329–336.
5. Traina F, Visconte V, Elson P, Tabarroki A, Jankowska AM, Hasrouni E *et al*. Impact of molecular mutations on treatment response to DNMT inhibitors in myelodysplasia and related neoplasms. *Leukemia* 2014; **28**: 78–87.

**CMML:**

1. Patnaik MM, Tefferi, A. Cytogenetic and molecular abnormalities in chronic myelomonocytic leukemia. *Blood Cancer J* 2016; **6**: e393.
2. Atlas of Genetics and Cytogenetics in Oncology and Haematology. Chronic Myelomonocytic Leukemia (CMML). 2013. <http://atlasgeneticsoncology.org/Anomalies/CMMLID1098.html.>
3. Valent P, Orazi A, Savona MR, Patnaik MM, Onida F, Van De Loosdrecht AA *et al*. Proposed diagnostic criteria for classical chronic myelomonocytic leukemia (CMML), CMML variants and pre-CMML conditions. *Haematologica* 2019; **104**: 1935–1949.
4. Reinig, E. et al. Targeted next-generation sequencing in myelodysplastic syndrome and chronic myelomonocytic leukemia aids diagnosis in challenging cases and identifies frequent spliceosome mutations in transformed acute myeloid leukemia. *Am J Clin Pathol* 2016; **145**: 497–506.

**AML:**

1. The Cancer Genome Atlas Research Network. Genomic and epigenomic landscapes of adult de novo acute myeloid leukemia. *N Engl J Med* 2013; **368**: 2059–2074.
2. Metzeler KH, Herold T, Rothenberg-Thurley M, Amler S, Sauerland MC, Görlich D *et al*. Spectrum and prognostic relevance of driver gene mutations in acute myeloid leukemia. *Blood* 2016; **128**: 686–698.
3. Pashka P, Schlenk RF, Gaidzik VI, Herzig JK, Aulityzky T, Bullinger L *et al*. ASXL1 mutations in younger adult patients with acute myeloid leukemia: a study by the German-Austrian Acute Myeloid Leukemia Study Group. *Haematologica* 2015; **100**: 324–330.
4. Papaemmanuil E, Gerstung M, Bullinger L, Gaidzik VI, Paschka P, Roberts ND *et al*. Genomic classification and prognosis in acute myeloid leukemia. *N Engl J Med* 2016; **374**: 2209–2221.

**Supplementary Fig. 9 Mutation status and frequency of poor prognostic and frequently mutated genes and carrier response rate per treatment arm and indication.**

Next generation sequencing data was derived from 96 samples of which 88 were from response-evaluable patients. FMS-like tyrosine kinase 3–internal tandem duplication mutations were further confirmed by polymerase chain reaction. Mutational status and frequency of poor prognostic and frequently mutated genes and carrier response rate in the higher-risk MDS (**a**), higher-risk CMML (**b**) and LB-AML (**c**) cohorts are shown. Each column represents a single patient and each row represents a single gene. Comparative analysis of mutation frequency distribution and carrier response rate per study arm are described. Responders in **a** and **b** include complete remission + partial remission + hematologic improvement. Responders in **c** include complete remission + complete remission with incomplete blood count recovery + partial remission. Presence and absence of a mutation in any gene is denoted in red and white, respectively.


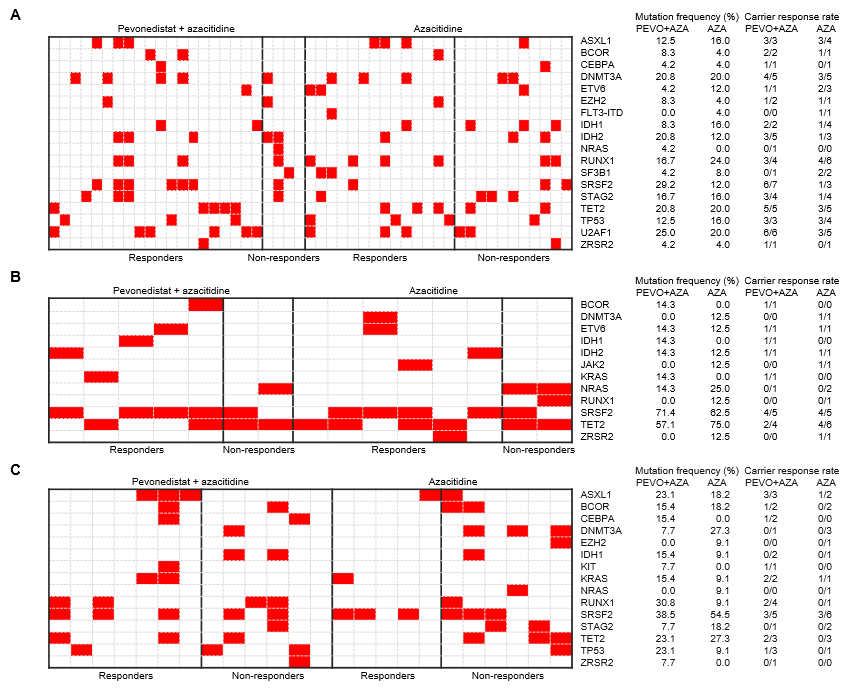


*AZA* azacitidine, *CMML* chronic myelomonocytic leukemia, *LB-AML* low-blast acute myeloid leukemia, *MDS* myelodysplastic syndromes, *PEVO* pevonedistat.

**Supplementary Fig. 10 Overall response rate in patients with higher-risk myelodysplastic syndromes harboring poor prognostic mutations.**Comparative analysis of overall response rate between study arms in patients in the higher-risk myelodysplastic syndromes cohort harboring poor prognostic mutations. Number of patients achieving a response (complete remission + partial remission + hematologic improvement) out of total number of patients harboring a particular mutation is described.


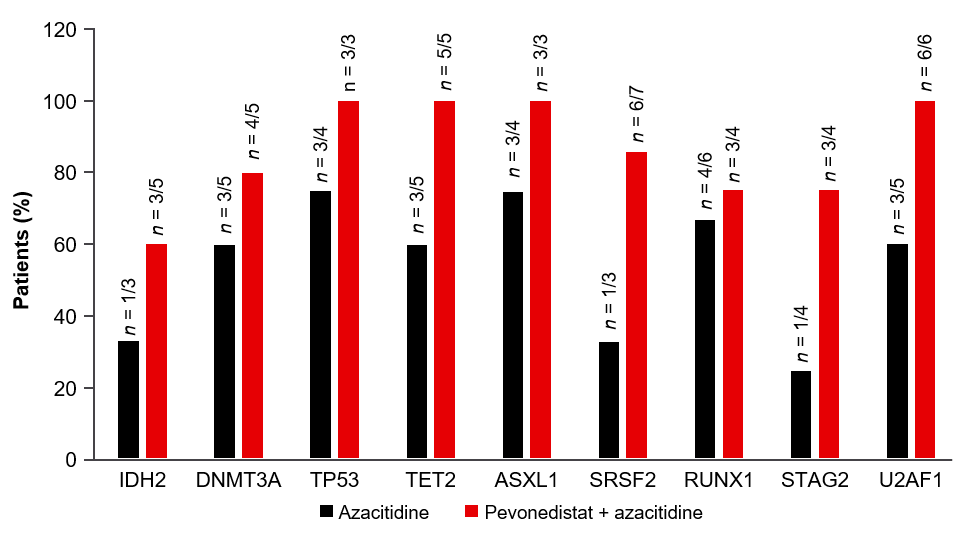


**Supplementary Table 1** Patient demographics and baseline characteristics.

|  | **Pevonedistat+ azacitidine**  ***n* = 58** | **Azacitidine  alone**  ***n* = 62** | **Total  *N* = 120** |
| --- | --- | --- | --- |
| Median age, years (range) | 74 (47–91) | 71 (34–84) | 72 (34–91) |
| Male/female, *n* (%) | 42 (72)/16 (28) | 41 (66)/21 (34) | 83 (69)/37 (31) |
| **Higher-risk MDS** | ***n* = 32** | ***n* = 35** | ***n* = 67** |
| Disease type, *n* (%)  De novo  Secondary | 32 (100)  0 | 34 (97)  1 (3) | 66 (99)  1 (1) |
| WHO Tumor Classification, *n* (%)  Refractory anemia with excess blasts-1  Refractory anemia with excess blasts-2  Not available  Missing | 12 (38)  18 (56)  1 (3)  1 | 15 (43)  18 (51)  1 (3)  1 | 27 (40)  36 (54)  2 (3)  2 |
| IPSS-R category, *n* (%)  Intermediate risk  High risk  Very high risk | 11 (34)  11 (34)  10 (31) | 9 (26)  10 (29)  16 (46) | 20 (30)  21 (31)  26 (39) |
| ECOG PS, *n* (%)  0  1  2 | 15 (47)  15 (47)  2 (6) | 18 (51)  16 (46)  1 (3) | 33 (49)  31 (46)  3 (4) |
| Median modified Charlson Comorbidity Index, (range) | 0 (0–6) | 0 (0–3) | 0 (0–6) |
| Median time from initial diagnosis, months (range) | 2.3 (0.2–58.4) | 1.7 (0.6–79.1) | 1.7 (0.2–79.1) |
| **Higher-risk CMML** | ***n* = 9** | ***n* = 8** | ***n* = 17** |
| Disease type, *n* (%)  De novo | 9 (100) | 8 (100) | 17 (100) |
| WHO Tumor Classification, *n* (%)  CMML-1  CMML-2  Missing | 4 (44)  3 (33)  2 | 6 (75)  1 (13)  1 | 10 (59)  4 (24)  3 |
| IPSS-R category, *n* (%)  Intermediate risk  High risk  Very high risk | 4 (44)  4 (44)  1 (11) | 5 (63)  2 (25)  1 (13) | 9 (53)  6 (35)  2 (12) |
| ECOG PS, *n* (%)  0  1  2 | 3 (33)  5 (56)  1 (11) | 5 (63)  2 (25)  1 (13) | 8 (47)  7 (41)  2 (12) |
| Median modified Charlson Comorbidity Index, (range) | 1 (0–5) | 2 (0–5) | 2 (0–5) |
| Median time from initial diagnosis, months (range) | 5.65 (2.1–87.9) | 5.06 (1.1–69.4) | 5.65 (1.1–87.9) |
| **LB-AML** | ***n* = 17** | ***n* = 19** | ***n* = 36** |
| Disease type, *n* (%)  De novo  Secondary  Unknown | 11 (65)  5 (29)  1 (6) | 14 (74)  5 (26)  0 | 25 (69)  10 (28)  1 (3) |
| Revised WHO Classification, *n* (%)  AML with recurrent genetic abnormalities  AML with myelodysplasia-related changes  AML not otherwise specified  Other  Missing | 2 (12)  12 (71)  3 (18)  0  0 | 1 (5)  8 (42)  8 (42)  1 (5)  1 | 3 (8)  20 (56)  11 (31)  1 (3)  1 |
| European LeukemiaNet risk classification  Intermediate  Adverse  Not available  Missing | 1 (6)  10 (59)  1 (6)  5 (29) | 2 (11)  5 (26)  5 (26)  7 (37) | 3 (8)  15 (42)  6 (17)  12 (33) |
| ECOG PS, *n* (%)  0  1 | 9 (53)  8 (47) | 10 (53)  9 (47) | 19 (53)  17 (47) |
| Median time from initial diagnosis, months (range) | 0.9 (0.0–4.0) | 0.8 (0.0–3.0) | 0.9 (0.0–4.0) |

*AML* acute myeloid leukemia, *CMML* chronic myelomonocytic leukemia, *ECOG PS* Eastern Cooperative Oncology Group performance status, *IPSS-R* Revised International Prognostic Scoring System, *LB-AML* low-blast acute myeloid leukemia, *MDS* myelodysplastic syndromes, *WHO* World Health Organization.

**Supplementary Table 2** Objective response.

| *n* (%), unless stated otherwise | **Pevonedistat+ azacitidine** | **Azacitidine  alone** |
| --- | --- | --- |
| **Overall population (response-evaluable)** | ***n* = 55** | ***n* = 53** |
| Overall response (CR + CRi + PR + HI) | 39 (70.9) | 32 (60.4) |
| CR | 22 (40.0) | 16 (30.2) |
| CRi | 3 (5.5) | 4 (7.5) |
| PR | 3 (5.5) | 4 (7.5) |
| HI | 11 (20.0) | 8 (15.1) |
| Median duration of response, months (95% CI) | 20.6 (10.71–34.60) | 13.1 (12.62–NE) |
| Median time to first CR/CRi/PR, months (range) in responding patients | 3.68 (1.6–25.8) | 3.79 (1.7–13.2) |
| **Overall population (ITT)** | ***n* = 58** | ***n* = 62** |
| Overall response (CR + CRi + PR + HI) | 39 (67.2) | 32 (51.6) |
| CR | 22 (37.9) | 16 (25.8) |
| CRi | 3 (5.2) | 4 (6.5) |
| PR | 3 (5.2) | 4 (6.5) |
| HI | 11 (19.0) | 8 (12.9) |
| **Higher-risk MDS (response-evaluable)** | ***n* = 30** | ***n* = 29** |
| Overall response (CR + PR + HI) | 23 (79.3) | 17 (56.7) |
| CR | 15 (51.7) | 8 (26.7) |
| PR | 1 (3.4) | 4 (13.3) |
| HI | 7 (24.1) | 5 (16.7) |
| Median duration of response, months (95% CI) | 34.6 (11.53–34.60) | 13.1 (12.02–NE) |
| **Higher-risk MDS (ITT)** | ***n* = 32** | ***n* = 35** |
| Overall response (CR + PR + HI) | 23 (71.9) | 17 (48.6) |
| CR | 15 (46.9) | 8 (22.9) |
| PR | 1 (3.1) | 4 (11.4) |
| HI | 7 (21.9) | 5 (14.3) |
| **Higher-risk CMML (response-evaluable and ITT^a^)** | ***n* = 9** | ***n* = 8** |
| Overall response (CR + PR + HI) | 7 (77.8) | 6 (75.0) |
| CR | 3 (33.3) | 3 (37.5) |
| PR | 0 | 0 |
| HI | 4 (44.4) | 3 (37.5) |
| Median duration of response, months (95% CI) | 13.9 (9.23–NE) | NE (NE–NE) |
| **LB-AML (response-evaluable)** | ***n* = 17** | ***n* = 15** |
| Overall response (CR + CRi + PR) | 9 (52.9) | 9 (60.0) |
| CR | 4 (23.5) | 5 (33.3) |
| CRi | 3 (17.6) | 4 (26.7) |
| PR | 2 (11.8) | 0 |
| Median duration of response, months (95% CI) | 13.7 (8.08–NE) | 12.9 (2.99–NE) |
| **LB-AML (ITT)** | ***n* = 17** | ***n* = 19** |
| Overall response (CR + CRi + PR) | 9 (52.9) | 9 (47.4) |
| CR | 4 (23.5) | 5 (26.3) |
| CRi | 3 (17.6) | 4 (21.1) |
| PR | 2 (11.8) | 0 |

*CI* confidence interval, *CMML* chronic myelomonocytic leukemia, *CR* complete remission, *CRi* complete remission with incomplete blood count recovery, *HI* hematologic improvement, *ITT* intent-to-treat, *LB-AML* low-blast acute myeloid leukemia, *MDS* myelodysplastic syndromes, *NE* not evaluable, *PR* partial remission.

^a^All patients in the higher-risk CMML subgroup were response-evaluable.
