# Supplementary material for: Randomized phase 2 trial of pevonedistat plus azacitidine versus azacitidine for higher-risk MDS/CMML or low-blast AML
Source: Leukemia. 2021 Jan 22;35(7):2119–24. doi: 10.1038/s41375-021-01125-4 (PMC8257476; doi:10.1038/s41375-021-01125-4)
Supplement: Supplementary file 2 — Supplementary Figure 1 [file 41375_2021_1125_MOESM2_ESM.pptx]

## Slide 1
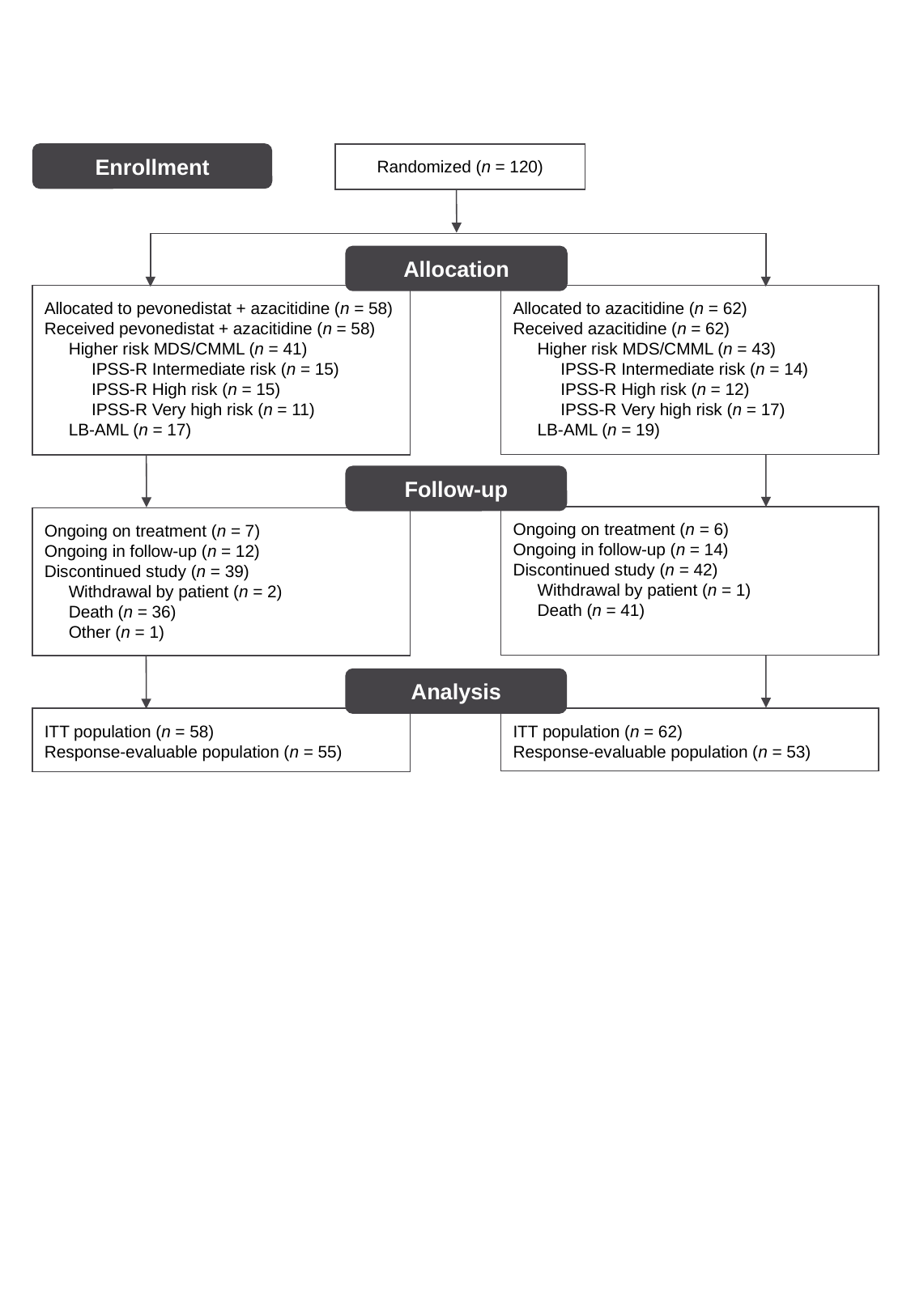

Enrollment
Randomized (n = 120)
Allocation
Allocated to pevonedistat + azacitidine (n = 58)
Received pevonedistat + azacitidine (n = 58)
Higher risk MDS/CMML (n = 41)
IPSS-R Intermediate risk (n = 15)
IPSS-R High risk (n = 15)
IPSS-R Very high risk (n = 11)
LB-AML (n = 17)
Allocated to azacitidine (n = 62)
Received azacitidine (n = 62)
Higher risk MDS/CMML (n = 43)
IPSS-R Intermediate risk (n = 14)
IPSS-R High risk (n = 12)
IPSS-R Very high risk (n = 17)
LB-AML (n = 19)
Follow-up
Ongoing on treatment (n = 6)
Ongoing in follow-up (n = 14)
Discontinued study (n = 42)
Withdrawal by patient (n = 1)
Death (n = 41)
Ongoing on treatment (n = 7)
Ongoing in follow-up (n = 12)
Discontinued study (n = 39)
Withdrawal by patient (n = 2)
Death (n = 36)
Other (n = 1)
Analysis
ITT population (n = 58)
Response-evaluable population (n = 55)
ITT population (n = 62)
Response-evaluable population (n = 53)
