# Supplementary material for: Randomized phase 2 trial of pevonedistat plus azacitidine versus azacitidine for higher-risk MDS/CMML or low-blast AML
Source: Leukemia. 2021 Jan 22;35(7):2119–24. doi: 10.1038/s41375-021-01125-4 (PMC8257476; doi:10.1038/s41375-021-01125-4)
Supplement: Supplementary file 3 — Supplementary Figure 2 [file 41375_2021_1125_MOESM3_ESM.pptx]

## Slide 1
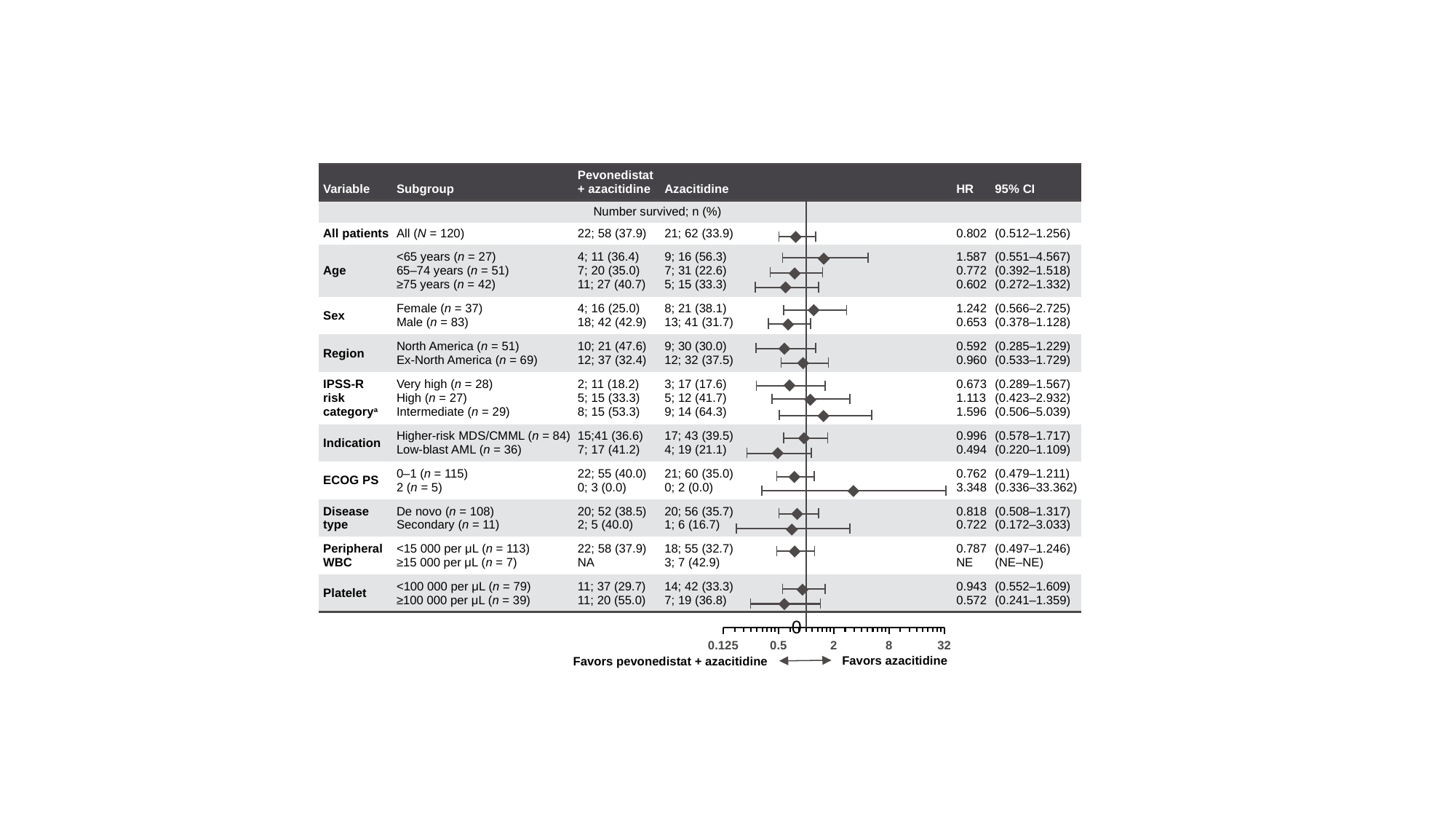

### Chart
| Category | | | |
|---|---|---|---|Favors azacitidine
Favors pevonedistat + azacitidine
| Variable | Subgroup | Pevonedistat + azacitidine | Azacitidine | | HR | 95% CI |
| --- | --- | --- | --- | --- | --- | --- |
| | | Number survived; n (%) | | | | |
| All patients | All (N = 120) | 22; 58 (37.9) | 21; 62 (33.9) | | 0.802 | (0.512–1.256) |
| Age | <65 years (n = 27) 65–74 years (n = 51) ≥75 years (n = 42) | 4; 11 (36.4) 7; 20 (35.0) 11; 27 (40.7) | 9; 16 (56.3) 7; 31 (22.6) 5; 15 (33.3) | | 1.587 0.772 0.602 | (0.551–4.567) (0.392–1.518) (0.272–1.332) |
| Sex | Female (n = 37) Male (n = 83) | 4; 16 (25.0) 18; 42 (42.9) | 8; 21 (38.1) 13; 41 (31.7) | | 1.242 0.653 | (0.566–2.725) (0.378–1.128) |
| Region | North America (n = 51) Ex-North America (n = 69) | 10; 21 (47.6) 12; 37 (32.4) | 9; 30 (30.0) 12; 32 (37.5) | | 0.592 0.960 | (0.285–1.229) (0.533–1.729) |
| IPSS-R risk categorya | Very high (n = 28) High (n = 27) Intermediate (n = 29) | 2; 11 (18.2) 5; 15 (33.3) 8; 15 (53.3) | 3; 17 (17.6) 5; 12 (41.7) 9; 14 (64.3) | | 0.673 1.113 1.596 | (0.289–1.567) (0.423–2.932) (0.506–5.039) |
| Indication | Higher-risk MDS/CMML (n = 84) Low-blast AML (n = 36) | 15;41 (36.6) 7; 17 (41.2) | 17; 43 (39.5) 4; 19 (21.1) | | 0.996 0.494 | (0.578–1.717) (0.220–1.109) |
| ECOG PS | 0–1 (n = 115) 2 (n = 5) | 22; 55 (40.0) 0; 3 (0.0) | 21; 60 (35.0) 0; 2 (0.0) | | 0.762 3.348 | (0.479–1.211) (0.336–33.362) |
| Disease type | De novo (n = 108) Secondary (n = 11) | 20; 52 (38.5) 2; 5 (40.0) | 20; 56 (35.7) 1; 6 (16.7) | | 0.818 0.722 | (0.508–1.317) (0.172–3.033) |
| Peripheral WBC | <15 000 per μL (n = 113) ≥15 000 per μL (n = 7) | 22; 58 (37.9) NA | 18; 55 (32.7) 3; 7 (42.9) | | 0.787 NE | (0.497–1.246) (NE–NE) |
| Platelet | <100 000 per μL (n = 79) ≥100 000 per μL (n = 39) | 11; 37 (29.7) 11; 20 (55.0) | 14; 42 (33.3) 7; 19 (36.8) | | 0.943 0.572 | (0.552–1.609) (0.241–1.359) |
