# Supplementary material for: Randomized phase 2 trial of pevonedistat plus azacitidine versus azacitidine for higher-risk MDS/CMML or low-blast AML
Source: Leukemia. 2021 Jan 22;35(7):2119–24. doi: 10.1038/s41375-021-01125-4 (PMC8257476; doi:10.1038/s41375-021-01125-4)
Supplement: Supplementary file 4 — Supplementary Figure 3 [file 41375_2021_1125_MOESM4_ESM.pptx]

## Slide 1
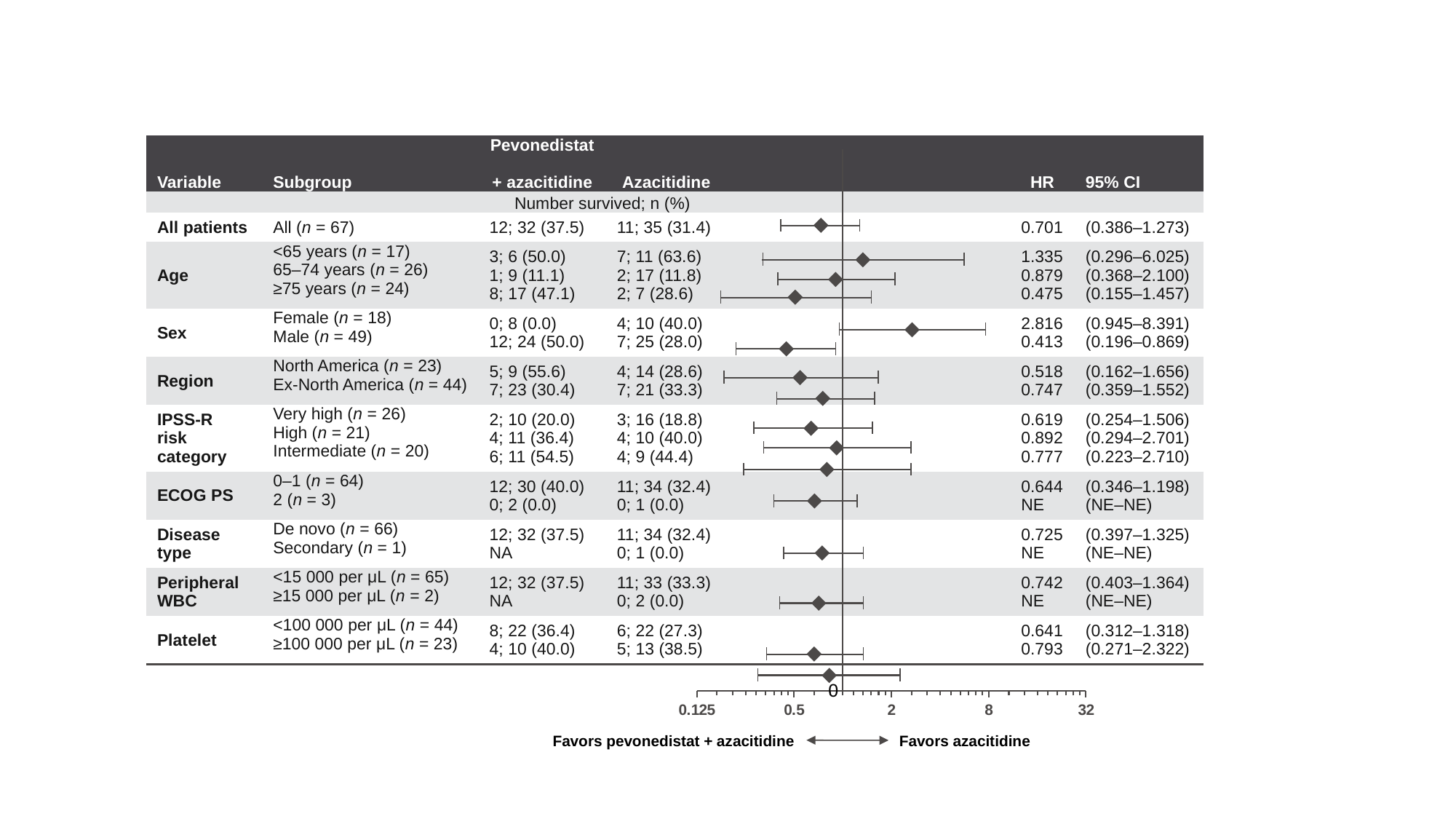

| Variable | Subgroup | Pevonedistat + azacitidine | Azacitidine | | HR | 95% CI |
| --- | --- | --- | --- | --- | --- | --- |
| | | Number survived; n (%) | | | | |
| All patients | All (n = 67) | 12; 32 (37.5) | 11; 35 (31.4) | | 0.701 | (0.386–1.273) |
| Age | <65 years (n = 17) 65–74 years (n = 26) ≥75 years (n = 24) | 3; 6 (50.0) 1; 9 (11.1) 8; 17 (47.1) | 7; 11 (63.6) 2; 17 (11.8) 2; 7 (28.6) | | 1.335 0.879 0.475 | (0.296–6.025) (0.368–2.100) (0.155–1.457) |
| Sex | Female (n = 18) Male (n = 49) | 0; 8 (0.0) 12; 24 (50.0) | 4; 10 (40.0) 7; 25 (28.0) | | 2.816 0.413 | (0.945–8.391) (0.196–0.869) |
| Region | North America (n = 23) Ex-North America (n = 44) | 5; 9 (55.6) 7; 23 (30.4) | 4; 14 (28.6) 7; 21 (33.3) | | 0.518 0.747 | (0.162–1.656) (0.359–1.552) |
| IPSS-R risk category | Very high (n = 26) High (n = 21) Intermediate (n = 20) | 2; 10 (20.0) 4; 11 (36.4) 6; 11 (54.5) | 3; 16 (18.8) 4; 10 (40.0) 4; 9 (44.4) | | 0.619 0.892 0.777 | (0.254–1.506) (0.294–2.701) (0.223–2.710) |
| ECOG PS | 0–1 (n = 64) 2 (n = 3) | 12; 30 (40.0) 0; 2 (0.0) | 11; 34 (32.4) 0; 1 (0.0) | | 0.644 NE | (0.346–1.198) (NE–NE) |
| Disease type | De novo (n = 66) Secondary (n = 1) | 12; 32 (37.5) NA | 11; 34 (32.4) 0; 1 (0.0) | | 0.725 NE | (0.397–1.325) (NE–NE) |
| Peripheral WBC | <15 000 per μL (n = 65) ≥15 000 per μL (n = 2) | 12; 32 (37.5) NA | 11; 33 (33.3) 0; 2 (0.0) | | 0.742 NE | (0.403–1.364) (NE–NE) |
| Platelet | <100 000 per μL (n = 44) ≥100 000 per μL (n = 23) | 8; 22 (36.4) 4; 10 (40.0) | 6; 22 (27.3) 5; 13 (38.5) | | 0.641 0.793 | (0.312–1.318) (0.271–2.322) |
### Chart
| Category | | | |
|---|---|---|---|
Favors pevonedistat + azacitidine
Favors azacitidine
