# Supplementary material for: Randomized phase 2 trial of pevonedistat plus azacitidine versus azacitidine for higher-risk MDS/CMML or low-blast AML
Source: Leukemia. 2021 Jan 22;35(7):2119–24. doi: 10.1038/s41375-021-01125-4 (PMC8257476; doi:10.1038/s41375-021-01125-4)
Supplement: Supplementary file 5 — Supplementary Figure 4 [file 41375_2021_1125_MOESM5_ESM.pptx]

## Slide 1
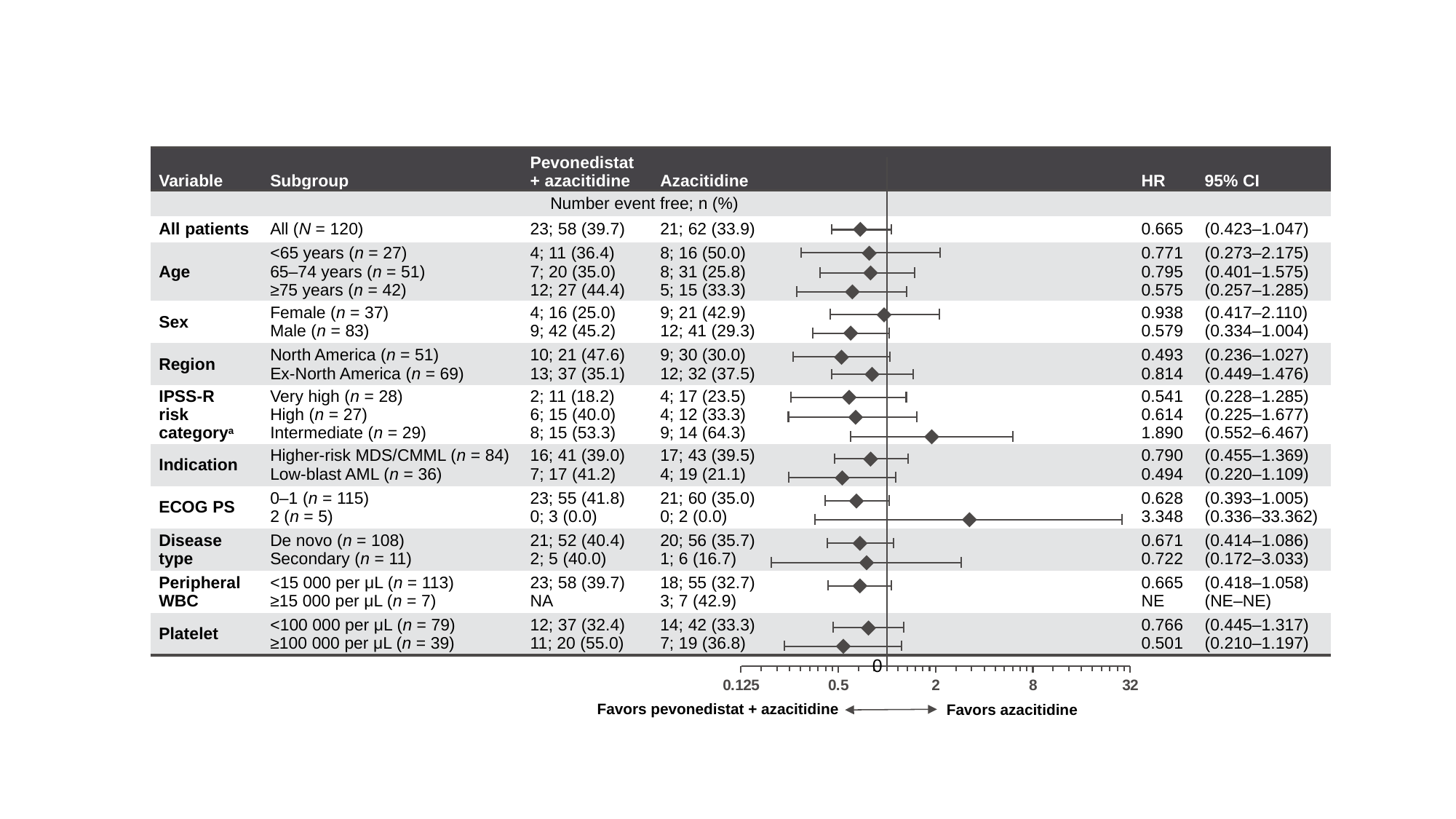

### Chart
| Category | | | |
|---|---|---|---|| Variable | Subgroup | Pevonedistat + azacitidine | Azacitidine | | HR | 95% CI |
| --- | --- | --- | --- | --- | --- | --- |
| | | Number event free; n (%) | | | | |
| All patients | All (N = 120) | 23; 58 (39.7) | 21; 62 (33.9) | | 0.665 | (0.423–1.047) |
| Age | <65 years (n = 27) 65–74 years (n = 51) ≥75 years (n = 42) | 4; 11 (36.4) 7; 20 (35.0) 12; 27 (44.4) | 8; 16 (50.0) 8; 31 (25.8) 5; 15 (33.3) | | 0.771 0.795 0.575 | (0.273–2.175) (0.401–1.575) (0.257–1.285) |
| Sex | Female (n = 37) Male (n = 83) | 4; 16 (25.0) 9; 42 (45.2) | 9; 21 (42.9) 12; 41 (29.3) | | 0.938 0.579 | (0.417–2.110) (0.334–1.004) |
| Region | North America (n = 51) Ex-North America (n = 69) | 10; 21 (47.6) 13; 37 (35.1) | 9; 30 (30.0) 12; 32 (37.5) | | 0.493 0.814 | (0.236–1.027) (0.449–1.476) |
| IPSS-R risk categorya | Very high (n = 28) High (n = 27) Intermediate (n = 29) | 2; 11 (18.2) 6; 15 (40.0) 8; 15 (53.3) | 4; 17 (23.5) 4; 12 (33.3) 9; 14 (64.3) | | 0.541 0.614 1.890 | (0.228–1.285) (0.225–1.677) (0.552–6.467) |
| Indication | Higher-risk MDS/CMML (n = 84) Low-blast AML (n = 36) | 16; 41 (39.0) 7; 17 (41.2) | 17; 43 (39.5) 4; 19 (21.1) | | 0.790 0.494 | (0.455–1.369) (0.220–1.109) |
| ECOG PS | 0–1 (n = 115) 2 (n = 5) | 23; 55 (41.8) 0; 3 (0.0) | 21; 60 (35.0) 0; 2 (0.0) | | 0.628 3.348 | (0.393–1.005) (0.336–33.362) |
| Disease type | De novo (n = 108) Secondary (n = 11) | 21; 52 (40.4) 2; 5 (40.0) | 20; 56 (35.7) 1; 6 (16.7) | | 0.671 0.722 | (0.414–1.086) (0.172–3.033) |
| Peripheral WBC | <15 000 per μL (n = 113) ≥15 000 per μL (n = 7) | 23; 58 (39.7) NA | 18; 55 (32.7) 3; 7 (42.9) | | 0.665 NE | (0.418–1.058) (NE–NE) |
| Platelet | <100 000 per μL (n = 79) ≥100 000 per μL (n = 39) | 12; 37 (32.4) 11; 20 (55.0) | 14; 42 (33.3) 7; 19 (36.8) | | 0.766 0.501 | (0.445–1.317) (0.210–1.197) |
Favors pevonedistat + azacitidine
Favors azacitidine
