# Supplementary material for: Randomized phase 2 trial of pevonedistat plus azacitidine versus azacitidine for higher-risk MDS/CMML or low-blast AML
Source: Leukemia. 2021 Jan 22;35(7):2119–24. doi: 10.1038/s41375-021-01125-4 (PMC8257476; doi:10.1038/s41375-021-01125-4)
Supplement: Supplementary file 6 — Supplementary Figure 5 [file 41375_2021_1125_MOESM6_ESM.pptx]

## Slide 1
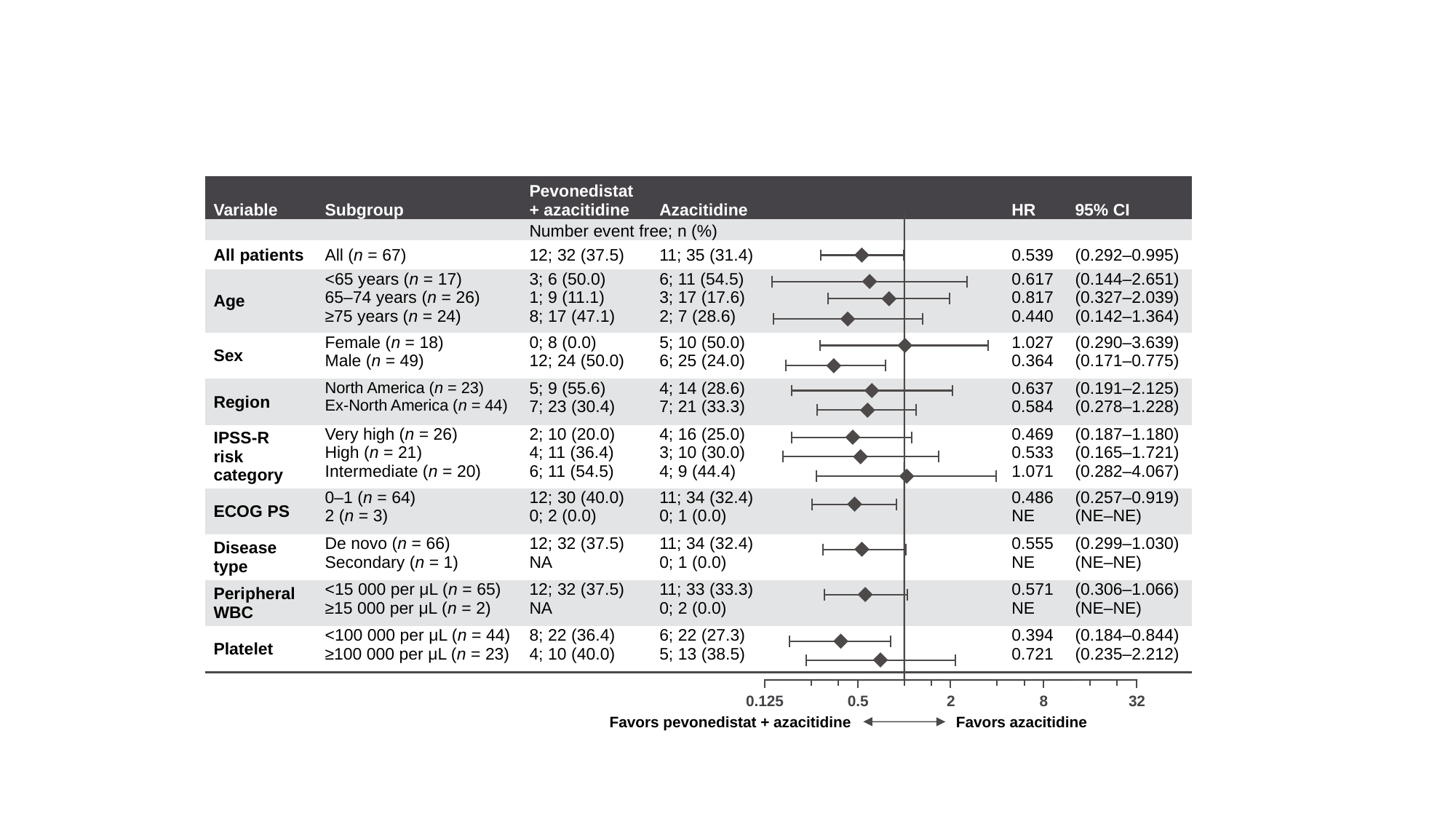

| Variable | Subgroup | Pevonedistat + azacitidine | Azacitidine | | HR | 95% CI |
| --- | --- | --- | --- | --- | --- | --- |
| | | Number event free; n (%) | | | | |
| All patients | All (n = 67) | 12; 32 (37.5) | 11; 35 (31.4) | | 0.539 | (0.292–0.995) |
| Age | <65 years (n = 17) 65–74 years (n = 26) ≥75 years (n = 24) | 3; 6 (50.0) 1; 9 (11.1) 8; 17 (47.1) | 6; 11 (54.5) 3; 17 (17.6) 2; 7 (28.6) | | 0.617 0.817 0.440 | (0.144–2.651) (0.327–2.039) (0.142–1.364) |
| Sex | Female (n = 18) Male (n = 49) | 0; 8 (0.0) 12; 24 (50.0) | 5; 10 (50.0) 6; 25 (24.0) | | 1.027 0.364 | (0.290–3.639) (0.171–0.775) |
| Region | North America (n = 23) Ex-North America (n = 44) | 5; 9 (55.6) 7; 23 (30.4) | 4; 14 (28.6) 7; 21 (33.3) | | 0.637 0.584 | (0.191–2.125) (0.278–1.228) |
| IPSS-R risk category | Very high (n = 26) High (n = 21) Intermediate (n = 20) | 2; 10 (20.0) 4; 11 (36.4) 6; 11 (54.5) | 4; 16 (25.0) 3; 10 (30.0) 4; 9 (44.4) | | 0.469 0.533 1.071 | (0.187–1.180) (0.165–1.721) (0.282–4.067) |
| ECOG PS | 0–1 (n = 64) 2 (n = 3) | 12; 30 (40.0) 0; 2 (0.0) | 11; 34 (32.4) 0; 1 (0.0) | | 0.486 NE | (0.257–0.919) (NE–NE) |
| Disease type | De novo (n = 66) Secondary (n = 1) | 12; 32 (37.5) NA | 11; 34 (32.4) 0; 1 (0.0) | | 0.555 NE | (0.299–1.030) (NE–NE) |
| Peripheral WBC | <15 000 per μL (n = 65) ≥15 000 per μL (n = 2) | 12; 32 (37.5) NA | 11; 33 (33.3) 0; 2 (0.0) | | 0.571 NE | (0.306–1.066) (NE–NE) |
| Platelet | <100 000 per μL (n = 44) ≥100 000 per μL (n = 23) | 8; 22 (36.4) 4; 10 (40.0) | 6; 22 (27.3) 5; 13 (38.5) | | 0.394 0.721 | (0.184–0.844) (0.235–2.212) |
0.125
0.5
2
8
32
Favors pevonedistat + azacitidine
Favors azacitidine
