# Supplementary material for: Randomized phase 2 trial of pevonedistat plus azacitidine versus azacitidine for higher-risk MDS/CMML or low-blast AML
Source: Leukemia. 2021 Jan 22;35(7):2119–24. doi: 10.1038/s41375-021-01125-4 (PMC8257476; doi:10.1038/s41375-021-01125-4)
Supplement: Supplementary file 7 — Supplementary Figure 6 [file 41375_2021_1125_MOESM7_ESM.pptx]

## Slide 1
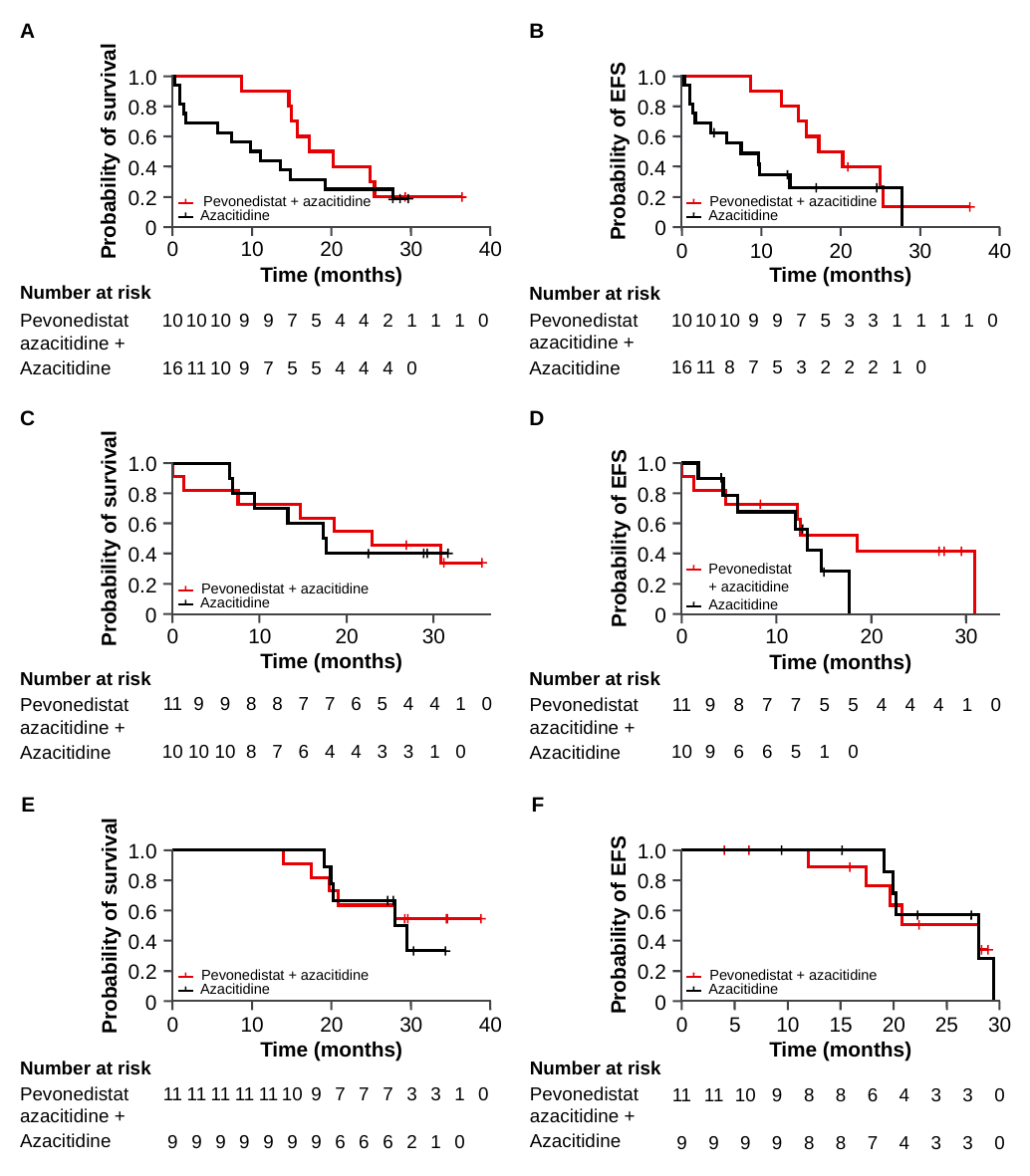

A
B
1.0
1.0
0.8
0.8
0.6
0.6
Probability of survival
Probability of EFS
0.4
0.4
0.2
0.2
Pevonedistat + azacitidine
Azacitidine
Pevonedistat + azacitidine
Azacitidine
0
0
0
10
20
30
40
0
10
20
30
40
Time (months)
Time (months)
Number at risk
Number at risk
Pevonedistatazacitidine +
10
10
10
9
9
7
5
3
3
1
1
1
1
0
10
10
10
9
9
7
5
4
4
2
1
1
1
0
Pevonedistatazacitidine +
16
11
8
7
5
3
2
2
2
1
0
16
11
10
9
7
5
5
4
4
4
0
Azacitidine
Azacitidine
C
D
1.0
1.0
0.8
0.8
0.6
0.6
Probability of survival
Probability of EFS
0.4
0.4
Pevonedistat
+ azacitidine
Azacitidine
0.2
0.2
Pevonedistat + azacitidine
Azacitidine
0
0
0
10
20
30
0
10
20
30
Time (months)
Time (months)
Number at risk
Number at risk
11
9
9
8
8
7
7
6
5
4
4
1
0
Pevonedistatazacitidine +
Pevonedistatazacitidine +
11
9
8
7
7
5
5
4
4
4
1
0
10
9
6
6
5
1
0
10
10
10
8
7
6
4
4
3
3
1
0
Azacitidine
Azacitidine
E
F
1.0
1.0
0.8
0.8
0.6
0.6
Probability of survival
Probability of EFS
0.4
0.4
0.2
0.2
Pevonedistat + azacitidine
Azacitidine
Pevonedistat + azacitidine
Azacitidine
0
0
0
10
20
30
40
0
5
10
15
20
25
30
Time (months)
Time (months)
Number at risk
Number at risk
Pevonedistatazacitidine +
Pevonedistatazacitidine +
11
11
11
11
11
10
9
7
7
7
3
3
1
0
11
11
10
9
8
8
6
4
3
3
0
Azacitidine
Azacitidine
9
9
9
9
9
9
9
6
6
6
2
1
0
9
9
9
9
8
8
7
4
3
3
0
