# Supplementary material for: Randomized phase 2 trial of pevonedistat plus azacitidine versus azacitidine for higher-risk MDS/CMML or low-blast AML
Source: Leukemia. 2021 Jan 22;35(7):2119–24. doi: 10.1038/s41375-021-01125-4 (PMC8257476; doi:10.1038/s41375-021-01125-4)
Supplement: Supplementary file 8 — Supplementary Figure 7 [file 41375_2021_1125_MOESM8_ESM.pptx]

## Slide 1
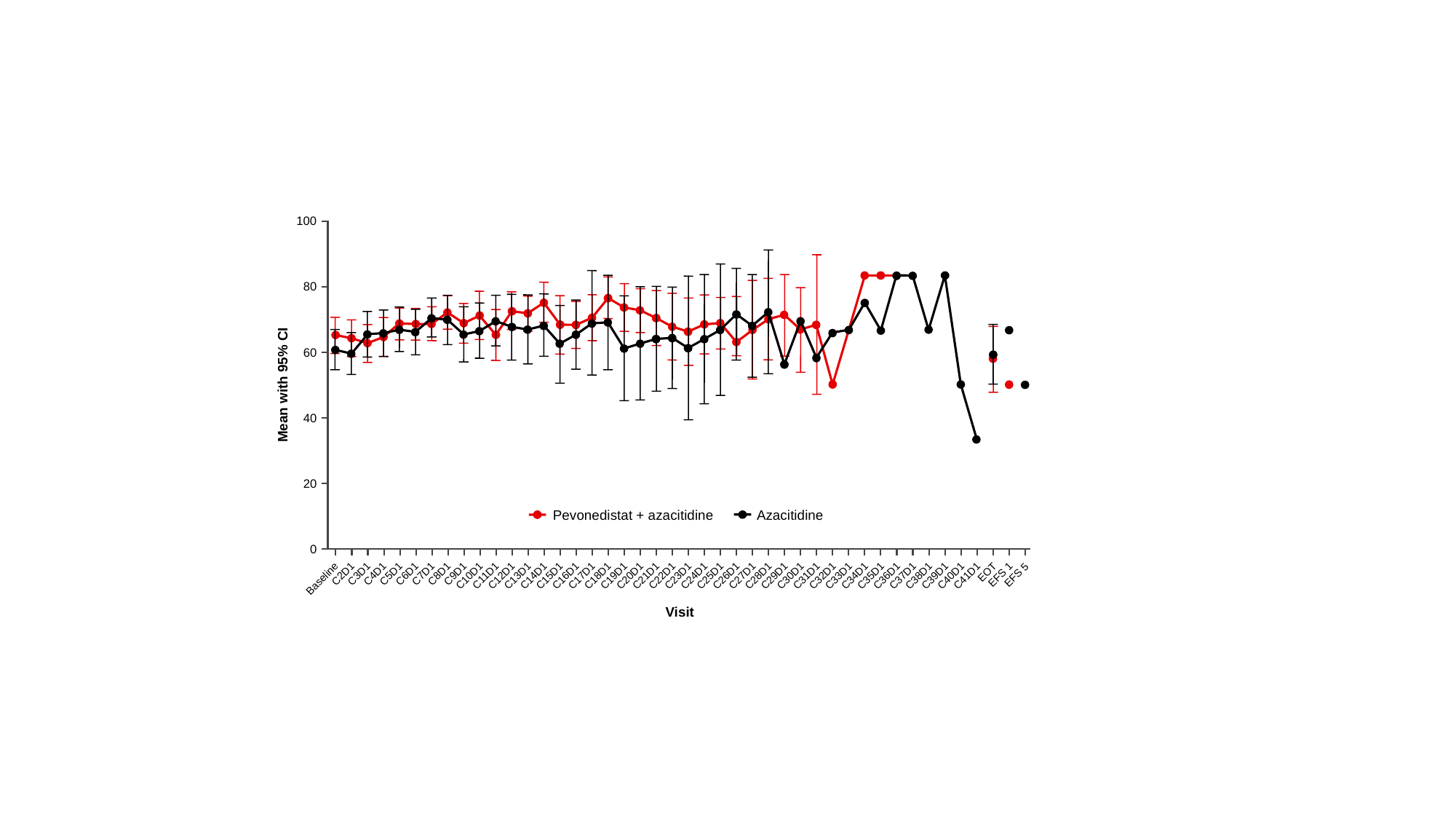

100
80
60
40
20
0
Mean with 95% CI
Pevonedistat + azacitidine
Azacitidine
Baseline
C2D1
C4D1
C6D1
C12D1
C14D1
C16D1
C8D1
C18D1
C22D1
C24D1
C26D1
C32D1
C34D1
C36D1
C3D1
C5D1
C7D1
C13D1
C15D1
C17D1
C28D1
C38D1
C9D1
C19D1
C23D1
C25D1
C27D1
C33D1
C35D1
C37D1
C10D1
C20D1
C29D1
C39D1
EOT
C11D1
C21D1
C30D1
C40D1
EFS 1
C31D1
C41D1
EFS 5
Visit
