# Supplementary material for: Randomized phase 2 trial of pevonedistat plus azacitidine versus azacitidine for higher-risk MDS/CMML or low-blast AML
Source: Leukemia. 2021 Jan 22;35(7):2119–24. doi: 10.1038/s41375-021-01125-4 (PMC8257476; doi:10.1038/s41375-021-01125-4)
Supplement: Supplementary file 9 — Supplementary Figure 8 [file 41375_2021_1125_MOESM9_ESM.pptx]

## Slide 1
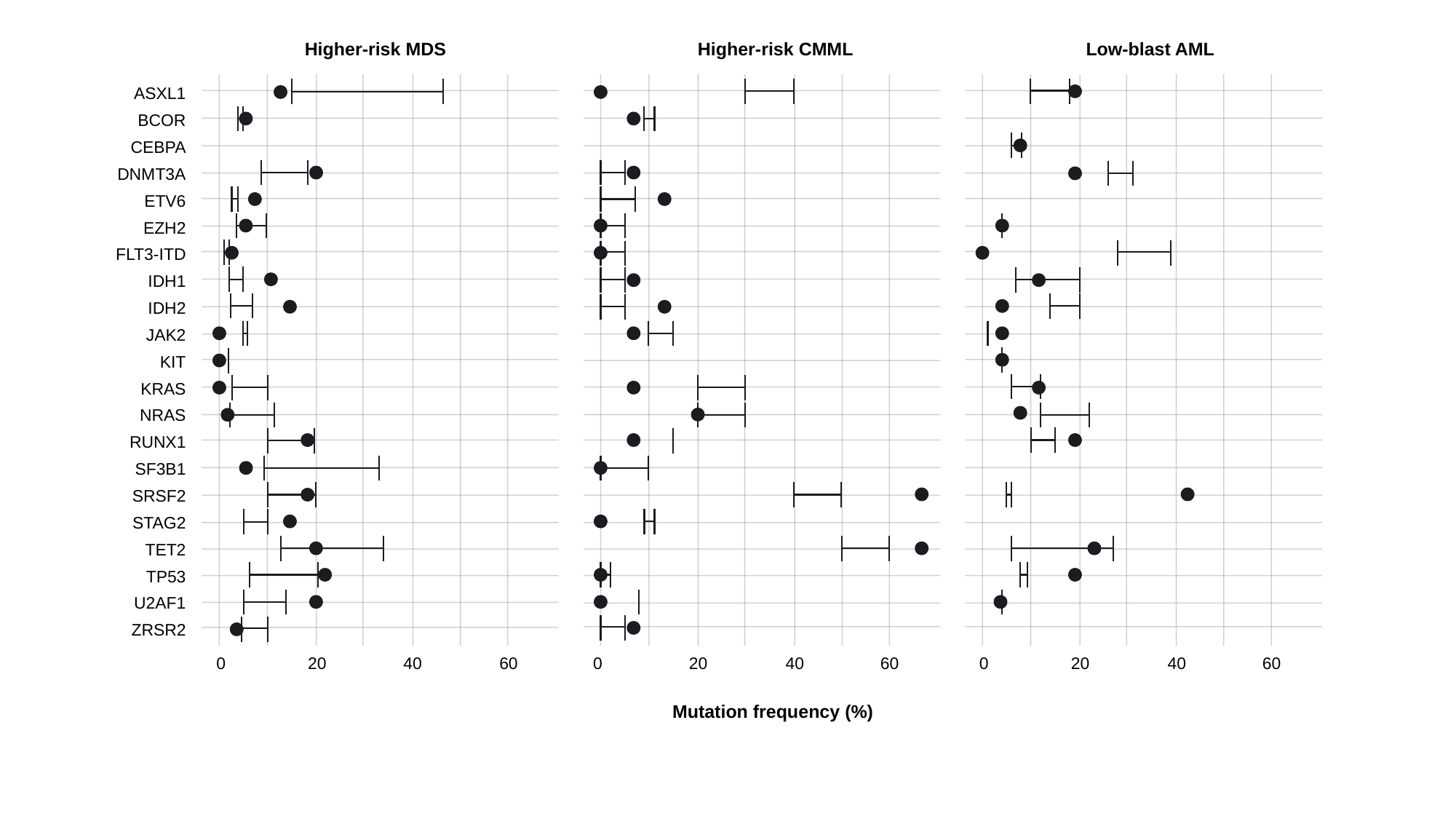

Low-blast AML
Higher-risk CMML
Higher-risk MDS
ASXL1
BCOR
CEBPA
DNMT3A
ETV6
EZH2
FLT3-ITD
IDH1
IDH2
JAK2
KIT
KRAS
NRAS
RUNX1
SF3B1
SRSF2
STAG2
TET2
TP53
U2AF1
ZRSR2
0
20
40
60
0
20
40
60
0
20
40
60
Mutation frequency (%)
