# Supplementary material for: Randomized phase 2 trial of pevonedistat plus azacitidine versus azacitidine for higher-risk MDS/CMML or low-blast AML
Source: Leukemia. 2021 Jan 22;35(7):2119–24. doi: 10.1038/s41375-021-01125-4 (PMC8257476; doi:10.1038/s41375-021-01125-4)
Supplement: Supplementary file 10 — Supplementary Figure 9 [file 41375_2021_1125_MOESM10_ESM.pptx]

## Slide 1
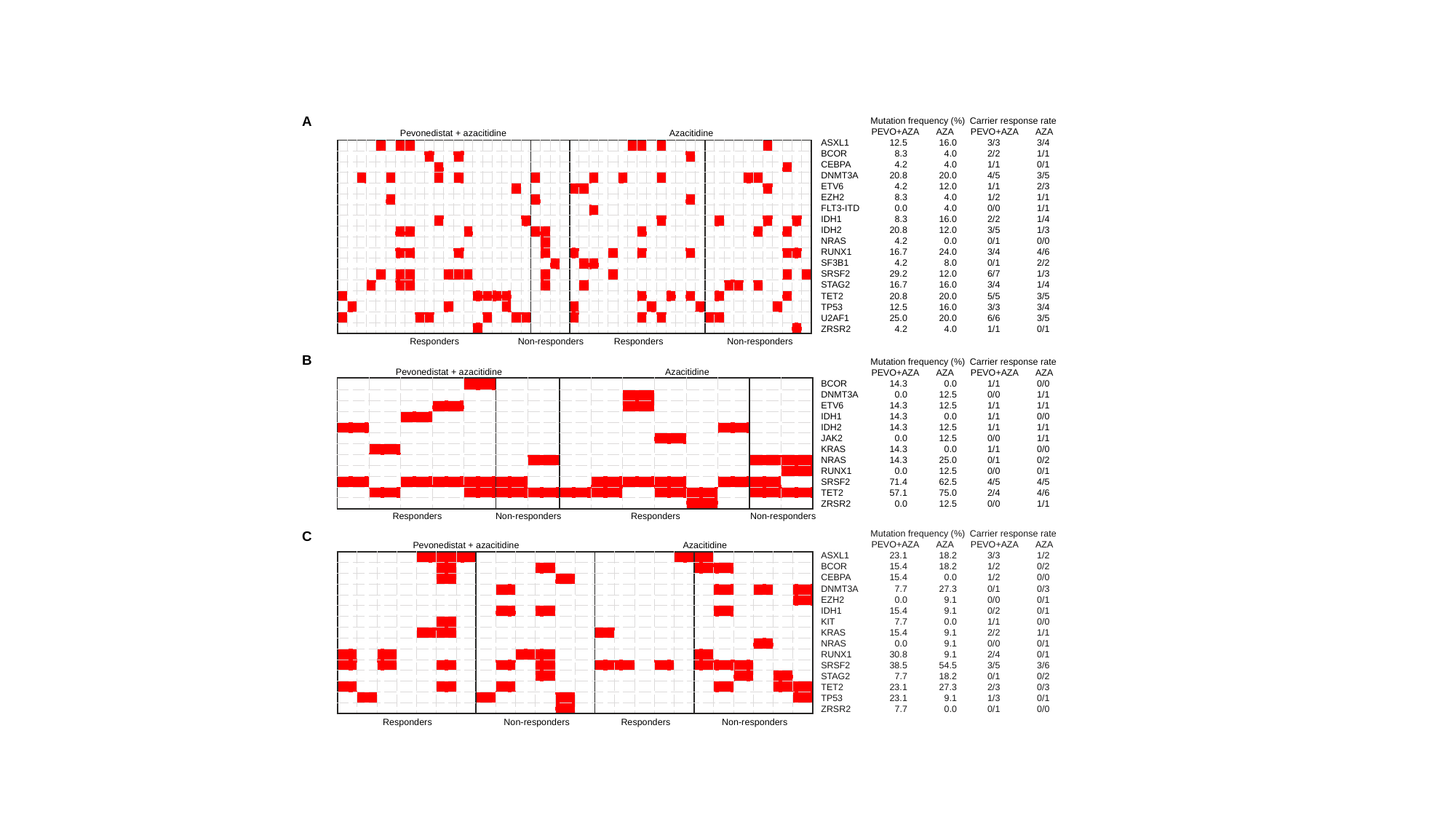

A
| | Mutation frequency (%) | | Carrier response rate | |
| --- | --- | --- | --- | --- |
| | PEVO+AZA | AZA | PEVO+AZA | AZA |
| ASXL1 | 12.5 | 16.0 | 3/3 | 3/4 |
| BCOR | 8.3 | 4.0 | 2/2 | 1/1 |
| CEBPA | 4.2 | 4.0 | 1/1 | 0/1 |
| DNMT3A | 20.8 | 20.0 | 4/5 | 3/5 |
| ETV6 | 4.2 | 12.0 | 1/1 | 2/3 |
| EZH2 | 8.3 | 4.0 | 1/2 | 1/1 |
| FLT3-ITD | 0.0 | 4.0 | 0/0 | 1/1 |
| IDH1 | 8.3 | 16.0 | 2/2 | 1/4 |
| IDH2 | 20.8 | 12.0 | 3/5 | 1/3 |
| NRAS | 4.2 | 0.0 | 0/1 | 0/0 |
| RUNX1 | 16.7 | 24.0 | 3/4 | 4/6 |
| SF3B1 | 4.2 | 8.0 | 0/1 | 2/2 |
| SRSF2 | 29.2 | 12.0 | 6/7 | 1/3 |
| STAG2 | 16.7 | 16.0 | 3/4 | 1/4 |
| TET2 | 20.8 | 20.0 | 5/5 | 3/5 |
| TP53 | 12.5 | 16.0 | 3/3 | 3/4 |
| U2AF1 | 25.0 | 20.0 | 6/6 | 3/5 |
| ZRSR2 | 4.2 | 4.0 | 1/1 | 0/1 |
Pevonedistat + azacitidine
Azacitidine
| | | | | | | | | | | | | | | | | | | | | | | | | | | | | | | | | | | | | | | | | | | | | | | | | |
| --- | --- | --- | --- | --- | --- | --- | --- | --- | --- | --- | --- | --- | --- | --- | --- | --- | --- | --- | --- | --- | --- | --- | --- | --- | --- | --- | --- | --- | --- | --- | --- | --- | --- | --- | --- | --- | --- | --- | --- | --- | --- | --- | --- | --- | --- | --- | --- | --- |
| | | | | | | | | | | | | | | | | | | | | | | | | | | | | | | | | | | | | | | | | | | | | | | | | |
| | | | | | | | | | | | | | | | | | | | | | | | | | | | | | | | | | | | | | | | | | | | | | | | | |
| | | | | | | | | | | | | | | | | | | | | | | | | | | | | | | | | | | | | | | | | | | | | | | | | |
| | | | | | | | | | | | | | | | | | | | | | | | | | | | | | | | | | | | | | | | | | | | | | | | | |
| | | | | | | | | | | | | | | | | | | | | | | | | | | | | | | | | | | | | | | | | | | | | | | | | |
| | | | | | | | | | | | | | | | | | | | | | | | | | | | | | | | | | | | | | | | | | | | | | | | | |
| | | | | | | | | | | | | | | | | | | | | | | | | | | | | | | | | | | | | | | | | | | | | | | | | |
| | | | | | | | | | | | | | | | | | | | | | | | | | | | | | | | | | | | | | | | | | | | | | | | | |
| | | | | | | | | | | | | | | | | | | | | | | | | | | | | | | | | | | | | | | | | | | | | | | | | |
| | | | | | | | | | | | | | | | | | | | | | | | | | | | | | | | | | | | | | | | | | | | | | | | | |
| | | | | | | | | | | | | | | | | | | | | | | | | | | | | | | | | | | | | | | | | | | | | | | | | |
| | | | | | | | | | | | | | | | | | | | | | | | | | | | | | | | | | | | | | | | | | | | | | | | | |
| | | | | | | | | | | | | | | | | | | | | | | | | | | | | | | | | | | | | | | | | | | | | | | | | |
| | | | | | | | | | | | | | | | | | | | | | | | | | | | | | | | | | | | | | | | | | | | | | | | | |
| | | | | | | | | | | | | | | | | | | | | | | | | | | | | | | | | | | | | | | | | | | | | | | | | |
| | | | | | | | | | | | | | | | | | | | | | | | | | | | | | | | | | | | | | | | | | | | | | | | | |
| | | | | | | | | | | | | | | | | | | | | | | | | | | | | | | | | | | | | | | | | | | | | | | | | |
Responders
Non-responders
Responders
Non-responders
B
| | Mutation frequency (%) | | Carrier response rate | |
| --- | --- | --- | --- | --- |
| | PEVO+AZA | AZA | PEVO+AZA | AZA |
| BCOR | 14.3 | 0.0 | 1/1 | 0/0 |
| DNMT3A | 0.0 | 12.5 | 0/0 | 1/1 |
| ETV6 | 14.3 | 12.5 | 1/1 | 1/1 |
| IDH1 | 14.3 | 0.0 | 1/1 | 0/0 |
| IDH2 | 14.3 | 12.5 | 1/1 | 1/1 |
| JAK2 | 0.0 | 12.5 | 0/0 | 1/1 |
| KRAS | 14.3 | 0.0 | 1/1 | 0/0 |
| NRAS | 14.3 | 25.0 | 0/1 | 0/2 |
| RUNX1 | 0.0 | 12.5 | 0/0 | 0/1 |
| SRSF2 | 71.4 | 62.5 | 4/5 | 4/5 |
| TET2 | 57.1 | 75.0 | 2/4 | 4/6 |
| ZRSR2 | 0.0 | 12.5 | 0/0 | 1/1 |
Pevonedistat + azacitidine
Azacitidine
| | | | | | | | | | | | | | | |
| --- | --- | --- | --- | --- | --- | --- | --- | --- | --- | --- | --- | --- | --- | --- |
| | | | | | | | | | | | | | | |
| | | | | | | | | | | | | | | |
| | | | | | | | | | | | | | | |
| | | | | | | | | | | | | | | |
| | | | | | | | | | | | | | | |
| | | | | | | | | | | | | | | |
| | | | | | | | | | | | | | | |
| | | | | | | | | | | | | | | |
| | | | | | | | | | | | | | | |
| | | | | | | | | | | | | | | |
| | | | | | | | | | | | | | | |
Responders
Non-responders
Responders
Non-responders
C
| | Mutation frequency (%) | | Carrier response rate | |
| --- | --- | --- | --- | --- |
| | PEVO+AZA | AZA | PEVO+AZA | AZA |
| ASXL1 | 23.1 | 18.2 | 3/3 | 1/2 |
| BCOR | 15.4 | 18.2 | 1/2 | 0/2 |
| CEBPA | 15.4 | 0.0 | 1/2 | 0/0 |
| DNMT3A | 7.7 | 27.3 | 0/1 | 0/3 |
| EZH2 | 0.0 | 9.1 | 0/0 | 0/1 |
| IDH1 | 15.4 | 9.1 | 0/2 | 0/1 |
| KIT | 7.7 | 0.0 | 1/1 | 0/0 |
| KRAS | 15.4 | 9.1 | 2/2 | 1/1 |
| NRAS | 0.0 | 9.1 | 0/0 | 0/1 |
| RUNX1 | 30.8 | 9.1 | 2/4 | 0/1 |
| SRSF2 | 38.5 | 54.5 | 3/5 | 3/6 |
| STAG2 | 7.7 | 18.2 | 0/1 | 0/2 |
| TET2 | 23.1 | 27.3 | 2/3 | 0/3 |
| TP53 | 23.1 | 9.1 | 1/3 | 0/1 |
| ZRSR2 | 7.7 | 0.0 | 0/1 | 0/0 |
Pevonedistat + azacitidine
Azacitidine
| | | | | | | | | | | | | | | | | | | | | | | | |
| --- | --- | --- | --- | --- | --- | --- | --- | --- | --- | --- | --- | --- | --- | --- | --- | --- | --- | --- | --- | --- | --- | --- | --- |
| | | | | | | | | | | | | | | | | | | | | | | | |
| | | | | | | | | | | | | | | | | | | | | | | | |
| | | | | | | | | | | | | | | | | | | | | | | | |
| | | | | | | | | | | | | | | | | | | | | | | | |
| | | | | | | | | | | | | | | | | | | | | | | | |
| | | | | | | | | | | | | | | | | | | | | | | | |
| | | | | | | | | | | | | | | | | | | | | | | | |
| | | | | | | | | | | | | | | | | | | | | | | | |
| | | | | | | | | | | | | | | | | | | | | | | | |
| | | | | | | | | | | | | | | | | | | | | | | | |
| | | | | | | | | | | | | | | | | | | | | | | | |
| | | | | | | | | | | | | | | | | | | | | | | | |
| | | | | | | | | | | | | | | | | | | | | | | | |
| | | | | | | | | | | | | | | | | | | | | | | | |
Responders
Non-responders
Responders
Non-responders
