# Supplementary material for: Randomized phase 2 trial of pevonedistat plus azacitidine versus azacitidine for higher-risk MDS/CMML or low-blast AML
Source: Leukemia. 2021 Jan 22;35(7):2119–24. doi: 10.1038/s41375-021-01125-4 (PMC8257476; doi:10.1038/s41375-021-01125-4)
Supplement: Supplementary file 11 — Supplementary Figure 10 [file 41375_2021_1125_MOESM11_ESM.pptx]

## Slide 1
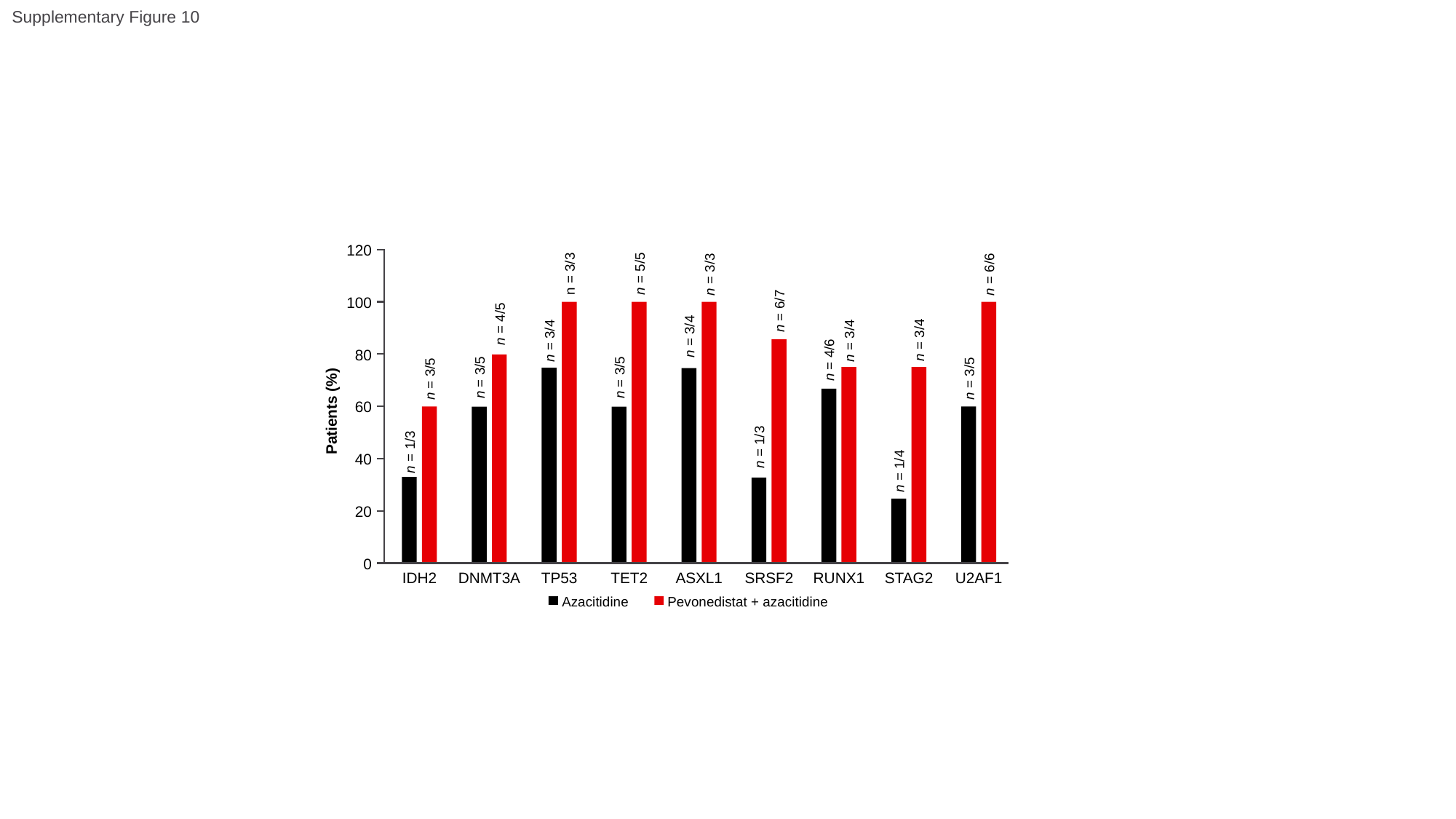

Supplementary Figure 10
120
100
80
60
40
20
0
n = 5/5
n = 3/5
TET2
n = 3/3
n = 3/4
TP53
n = 3/3
n = 3/4
ASXL1
n = 6/6
n = 3/5
U2AF1
n = 6/7
n = 1/3
SRSF2
n = 4/5
n = 3/5
DNMT3A
n = 3/4
n = 1/4
STAG2
n = 3/4
n = 4/6
RUNX1
n = 3/5
n = 1/3
IDH2
Patients (%)
Pevonedistat + azacitidine
Azacitidine
